# Effect of Bacille Calmette–Guérin vaccination on immune responses to SARS‐CoV‐2 and COVID‐19 vaccination

**DOI:** 10.1002/cti2.70023

**Published:** 2025-01-25

**Authors:** Nicole L Messina, Susie Germano, Amy W Chung, Carolien E van de Sandt, Natalie E Stevens, Lilith F Allen, Rhian Bonnici, Julio Croda, Claudio Counoupas, Branka Grubor‐Bauk, Ebene R Haycroft, Katherine Kedzierska, Ellie McDonald, Rebecca McElroy, Mihai G Netea, Boris Novakovic, Kirsten P Perrett, Laure F Pittet, Ruth A Purcell, Kanta Subbarao, James A Triccas, David J Lynn, Nigel Curtis, the BRACE Trial Consortium Group, Andrew Davidson, Kaya Gardiner, Amanda Gwee, Tenaya Jamieson, Nicole Messina, Thilanka Morawakage, Susan Perlen, Kirsten Perrett, Laure Pittet, Amber Sastry, Jia Wei Teo, Francesca Orsini, Katherine Lee, Cecilia Moore, Suzanna Vidmar, Laure Pittet, Rashida Ali, Ross Dunn, Peta Edler, Grace Gell, Casey Goodall, Richard Hall, Ann Krastev, Nathan La, Ellie McDonald, Nick McPhate, Thao Nguyen, Jack Ren, Luke Stevens, Nicole Messina, Ahmed Alamrousi, Rhian Bonnici, Thanh Dang, Jenny Hua, Monica Razmovska, Scott Reddiex, Xiaofang Wang, Jeremy Anderson, Kristy Azzopardi, Vicki Bennett‐Wood, Anna Czajko, Nadia Mazarakis, Conor McCafferty, Frances Oppedisano, Belinda Ortika, Casey Pell, Leena Spry, Ryan Toh, Sunitha Velagapudi, Amanda Vlahos, Ashleigh Wee‐Hee, Pedro Ramos, Karina De La Cruz, Dinusha Gamage, Anushka Karunanayake, Isabella Mezzetti, Benjamin Ong, Ronita Singh, Enoshini Sooriyarachchi, Suellen Nicholson, Natalie Cain, Rianne Brizuela, Han Huang, Veronica Abruzzo, Morgan Bealing, Patricia Bimboese, Kirsty Bowes, Emma Burrell, Joyce Chan, Jac Cushnahan, Hannah Elborough, Olivia Elkington, Kieran Fahey, Monique Fernandez, Catherine Flynn, Sarah Fowler, Marie Gentile Andrit, Bojana Gladanac, Catherine Hammond, Norine Ma, Sam Macalister, Emmah Milojevic, Jesutofunmi Mojeed, Jill Nguyen, Liz O'Donnell, Nadia Olivier, Isabelle Ooi, Stephanie Reynolds, Lisa Shen, Barb Sherry, Judith Spotswood, Jamie Wedderburn, Angela Younes, Donna Legge, Jason Bell, Jo Cheah, Annie Cobbledick, Kee Lim, Sonja Elia, Lynne Addlem, Anna Bourke, Clare Brophy, Nadine Henare, Narelle Jenkins, Francesca Machingaifa, Skye Miller, Kirsten Mitchell, Sigrid Pitkin, Kate Wall, Paola Villanueva, Nigel Crawford, Laure Pittet, Wendy Norton, Niki Tan, Thilakavathi Chengodu, Diane Dawson, Victoria Gordon, Tony Korman, Jess O'Bryan, Veronica Abruzzo, Sophie Agius, Samantha Bannister, Jess Bucholc, Alison Burns, Beatriz Camesella, John Carlin, Marianna Ciaverella, Maxwell Curtis, Stephanie Firth, Christina Guo, Matthew Hannan, Erin Hill, Sri Joshi, Katherine Lieschke, Megan Mathers, Sasha Odoi, Ashleigh Rak, Chris Richards, Leah Steve, Carolyn Stewart, Eva Sudbury, Helen Thomson, Emma Watts, Fiona Williams, Angela Young, Penny Glenn, Andrew Kaynes, Amandine Philippart De Floy, Sandy Buchanan, Thijs Sondag, Ivy Xie, Harriet Edmund, Bridie Byrne, Tom Keeble, Belle Ngien, Fran Noonan, Michelle Wearing‐Smith, Alison Clarke, Pemma Davies, Oliver Eastwood, Alric Ellinghaus, Rachid Ghieh, Zahra Hilton, Emma Jennings, Athina Kakkos, Iris Liang, Katie Nicol, Sally O'Callaghan, Helen Osman, Gowri Rajaram, Sophia Ratcliffe, Victoria Rayner, Ashleigh Salmon, Angela Scheppokat, Aimee Stevens, Rebekah Street, Nicholas Toogood, Nicholas Wood, Twinkle Bahaduri, Therese Baulman, Jennifer Byrne, Candace Carter, Mary Corbett, Aiken Dao, Maria Desylva, Andrew Dunn, Evangeline Gardiner, Rosemary Joyce, Rama Kandasamy, Craig Munns, Lisa Pelayo, Ketaki Sharma, Katrina Sterling, Caitlin Uren, Clinton Colaco, Mark Douglas, Kate Hamilton, Adam Bartlett, Brendan McMullan, Pamela Palasanthiran, Phoebe Williams, Justin Beardsley, Nikki Bergant, Renier Lagunday, Kristen Overton, Jeffrey Post, Yasmeen Al‐Hindawi, Sarah Barney, Anthony Byrne, Lee Mead, Marshall Plit, David Lynn, Saoirse Benson, Stephen Blake, Rochelle Botten, Tee Yee Chern, Georgina Eden, Liddy Griffith, Jane James, Miriam Lynn, Angela Markow, Domenic Sacca, Natalie Stevens, Steve Wesselingh, Catriona Doran, Simone Barry, Alice Sawka, Sue Evans, Louise Goodchild, Christine Heath, Meredith Krieg, Helen Marshall, Mark McMillan, Mary Walker, Peter Richmond, Nelly Amenyogbe, Christina Anthony, Annabelle Arnold, Beth Arrowsmith, Rym Ben‐Othman, Sharon Clark, Jemma Dunnill, Nat Eiffler, Krist Ewe, Carolyn Finucane, Lorraine Flynn, Camille Gibson, Lucy Hartnell, Elysia Hollams, Heidi Hutton, Lance Jarvis, Jane Jones, Jan Jones, Karen Jones, Jennifer Kent, Tobias Kollmann, Debbie Lalich, Wenna Lee, Rachel Lim, Sonia McAlister, Fiona McDonald, Andrea Meehan, Asma Minhaj, Lisa Montgomery, Melissa O'Donnell, Jaslyn Ong, Joanne Ong, Kimberley Parkin, Glady Perez, Catherine Power, Shadie Rezazadeh, Holly Richmond, Sally Rogers, Nikki Schultz, Margaret Shave, Patrycja Skut, Lisa Stiglmayer, Alexandra Truelove, Ushma Wadia, Rachael Wallace, Justin Waring, Michelle England, Erin Latkovic, Laurens Manning, Susan Herrmann, Michaela Lucas, Marcus Lacerda, Paulo Henrique Andrade, Fabiane Bianca Barbosa, Dayanne Barros, Larissa Brasil, Ana Greyce Capella, Ramon Castro, Erlane Costa, Dilcimar de Souza, Maianne Dias, José Dias, Klenilson Ferreira, Paula Figueiredo, Thamires Freitas, Ana Carolina Furtado, Larissa Gama, Vanessa Godinho, Cintia Gouy, Daniele Hinojosa, Bruno Jardim, Tyane Jardim, Joel Junior, Augustto Lima, Bernardo Maia, Adriana Marins, Kelry Mazurega, Tercilene Medeiros, Rosangela Melo, Marinete Moraes, Elizandra Nascimento, Juliana Neves, Maria Gabriela Oliveira, Thais Oliveira, Ingrid Oliveira, Arthur Otsuka, Rayssa Paes, Handerson Pereira, Gabrielle Pereira, Christiane Prado, Evelyn Queiroz, Laleyska Rodrigues, Bebeto Rodrigues, Vanderson Sampaio, Anna Gabriela Santos, Daniel Santos, Tilza Santos, Evelyn Santos, Ariandra Sartim, Ana Beatriz Silva, Juliana Silva, Emanuelle Silva, Mariana Simão, Caroline Soares, Antonny Sousa, Alexandre Trindade, Fernando Val, Adria Vasconcelos, Heline Vasconcelos, Julio Croda, Carolinne Abreu, Katya Martinez Almeida, Camila Bitencourt de Andrade, Jhenyfer Thalyta Campos Angelo, Ghislaine Gonçalvez de Araújo Arcanjo, Bianca Maria Silva Menezes Arruda, Wellyngthon Espindola Ayala, Adelita Agripina Refosco Barbosa, Felipe Zampieri Vieira Batista, Fabiani de Morais Batista, Miriam de Jesus Costa, Mariana Garcia Croda, Lais Alves da Cruz, Roberta Carolina Pereira Diogo, Rodrigo Cezar Dutra Escobar, Iara Rodrigues Fernandes, Leticia Ramires Figueiredo, Leandro Galdino Cavalcanti Gonçalves, Sarita Lahdo, Joyce dos Santos Lencina, Guilherme Teodoro de Lima, Larissa Santos Matos, Bruna Tayara Leopoldina Meireles, Debora Quadros Moreira, Lilian Batista Silva Muranaka, Adriely de Oliveira, Karla Regina Warszawski de Oliveira, Matheus Vieira de Oliveira, Roberto Dias de Oliveira, Andrea Antonia Souza de Almeida dos Reis Pereira, Marco Puga, Caroliny Veron Ramos, Thaynara Haynara Souza da Rosa, Karla Lopes dos Santos, Claudinalva Ribeiro dos Santos, Dyenyffer Stéffany Leopoldina dos Santos, Karina Marques Santos, Paulo César Pereira da Silva, Paulo Victor Rocha da Silva, Débora dos Santos Silva, Patricia Vieira da Silva, Bruno Freitas da Rosa Soares, Mariana Gazzoni Sperotto, Mariana Mayumi Tadokoro, Daniel Tsuha, Hugo Miguel Ramos Vieira, Margareth Maria Pretti Dalcolmo, Cíntia Maria Lopes Alves da Paixão, Gabriela Corrêa E Castro, Simone Silva Collopy, Renato da Costa Silva, Samyra Almeida da Silveira, Alda Maria Da‐Cruz, Alessandra Maria da Silva Passos de Carvalho, Rita de Cássia Batista, Maria Luciana Silva De Freitas, Aline Gerhardt de Oliveira Ferreira, Ana Paula Conceição de Souza, Paola Cerbino Doblas, Ayla Alcoforado da Silva dos Santos, Vanessa Cristine de Moraes dos Santos, Glauce Dos Santos, Dayane Alves dos Santos Gomes, Anderson Lage Fortunato, Adriano Gomes‐Silva, Monique Pinto Gonçalves, Paulo Leandro Garcia Meireles Junior, Estela Martins da Costa Carvalho, Fernando do Couto Motta, Ligia Maria Olivo de Mendonça, Girlene dos Santos Pandine, Rosa Maria Plácido Pereira, Ivan Ramos Maia, Jorge Luiz da Rocha, João Victor Paiva Romano, Erica Fernandes da Silva, Marilda AgudoMendonça Teixeira de Siqueira, Ágatha Cristinne Prudêncio Soares, Marc Bonten, Sandra Franch Arroyo, Cristina Prat Aymerich, Henny Ophorst‐den Besten, Anna Boon, Karin M Brakke, Axel Janssen, Marijke AH Koopmans, Toos Lemmens, Titia Leurink, Engelien Septer‐Bijleveld, Kimberly Stadhouders, Darren Troeman, Marije van der Waal, Marjoleine van Opdorp, Nicolette van Sluis, Beatrijs Wolters, Jan Kluytmans, Jannie Romme, Wouter van den Bijllaardt, Linda van Mook, MML van Rijen, PMG Filius, Jet Gisolf, Frances Greven, Danique Huijbens, Robert Jan Hassing, RC Pon, Lieke Preijers, JH van Leusen, Harald Verheij, Wim Boersma, Evelien Brans, Paul Kloeg, Kitty Molenaar‐Groot, Nhat Khanh Nguyen, Nienke Paternotte, Anke Rol, Lida Stooper, Helga Dijkstra, Esther Eggenhuizen, Lucas Huijs, Simone Moorlag, Mihai Netea, Eva Pranger, Esther Taks, Jaap ten Oever, Rob ter Heine, Kitty Blauwendraat, Bob Meek, Isil Erkaya, Houda Harbech, Nienke Roescher, Rifka Peeters, Menno te Riele, Carmen Zhou, Esther Calbo, Cristina Badia Marti, Emma Triviño Palomares, Tomás Perez Porcuna, Anabel Barriocanal, Ana Maria Barriocanal, Irma Casas, Jose Dominguez, Maria Esteve, Alicia Lacoma, Irene Latorre, Gemma Molina, Barbara Molina, Antoni Rosell, Sandra Vidal, Lydia Barrera, Natalia Bustos, Ines Portillo Calderón, David Gutierrez Campos, Jose Manuel Carretero, Angel Dominguez Castellano, Renato Compagnone, Encarnacion Ramirez de Arellano, Almudena de la Serna, Maria Dolores del Toro Lopez, Marie‐Alix Clement Espindola, Ana Belen Martin Gutierrez, Alvaro Pascual Hernandez, Virginia Palomo Jiménez, Elisa Moreno, Nicolas Navarrete, Teresa Rodriguez Paño, Jesús Rodríguez‐Baño, Enriqueta Tristán, Maria Jose Rios Villegas, Atsegiñe Canga Garces, Erika Castro Amo, Raquel Coya Guerrero, Josune Goikoetxea, Leticia Jorge, Cristina Perez, María Carmen Fariñas Álvarez, Manuel Gutierrez Cuadra, Francisco Arnaiz de las Revillas Almajano, Pilar Bohedo Garcia, Teresa Giménez Poderos, Claudia González Rico, Blanca Sanchez, Olga Valero, Noelia Vega, John Campbell, Anna Barnes, Helen Catterick, Tim Cranston, Phoebe Dawe, Emily Fletcher, Liam Fouracre, Alison Gifford, Neil Gow, John Kirkwood, Christopher Martin, Amy McAndrew, Marcus Mitchell, Georgina Newman, Abby O'Connell, Jakob Onysk, Lynne Quinn, Shelley Rhodes, Samuel Stone, Lorrie Symons, Harry Tripp, Adilia Warris, Darcy Watkins, Bethany Whale, Alex Harding, Gemma Lockhart, Kate Sidaway‐Lee, John Campbell, Sam Hilton, Sarah Manton, Daniel Webber‐Rookes, Rachel Winder, James Moore, Freya Bateman, Michael Gibbons, Bridget Knight, Julie Moss, Sarah Statton, Josephine Studham, Lydia Hall, Will Moyle, Tamsin Vent

**Affiliations:** ^1^ Infectious Diseases Group, Infection, Immunity and Global Health Theme Murdoch Children's Research Institute Parkville VIC Australia; ^2^ Department of Paediatrics The University of Melbourne Parkville VIC Australia; ^3^ Department of Microbiology and Immunology, The Peter Doherty Institute for Infection and Immunity The University of Melbourne Parkville VIC Australia; ^4^ Precision Medicine Theme South Australian Health and Medical Research Institute Adelaide SA Australia; ^5^ Flinders Health and Medical Research Institute Flinders University Bedford Park SA Australia; ^6^ Universidade Federal de Mato Grosso do Sul‐UFMS Campo Grande MS Brazil; ^7^ Fiocruz Mato Grosso do Sul Fundação Oswaldo Cruz Campo Grande MS Brazil; ^8^ Department of Epidemiology of Microbial Diseases Yale School of Public Health New Haven CT USA; ^9^ School of Medical Sciences, Faculty of Medicine and Health The University of Sydney Camperdown NSW Australia; ^10^ Sydney Institute for Infectious Diseases and the Charles Perkins Centre The University of Sydney Camperdown NSW Australia; ^11^ Centre for Infection and Immunity Centenary Institute Camperdown NSW Australia; ^12^ Viral Immunology Group, Adelaide Medical School, Basil Hetzel Institute for Translational Health Research University of Adelaide Adelaide SA Australia; ^13^ Department of Internal Medicine and Radboud Institute for Molecular Life Sciences Radboud University Medical Center Nijmegen The Netherlands; ^14^ Department for Immunology and Metabolism, Life and Medical Sciences Institute University of Bonn Bonn Germany; ^15^ Molecular Immunity Group, Infection and Immunity Theme Murdoch Children's Research Institute Parkville VIC Australia; ^16^ Population Allergy Group Murdoch Children's Research Institute Parkville VIC Australia; ^17^ Department of Allergy and Immunology The Royal Children's Hospital Melbourne Parkville VIC Australia; ^18^ Immunology, Vaccinology, Rheumatology and Infectious Diseases Unit Geneva University Hospitals and Faculty of Medicine Geneva Switzerland; ^19^ Department of Infectious Diseases The Royal Children's Hospital Melbourne Parkville VIC Australia

**Keywords:** Bacille Calmette–Guérin (BCG) vaccine, COVID‐19, heterologous, immunity, immunomodulation

## Abstract

**Objectives:**

Bacille Calmette–Guérin (BCG) vaccination has off‐target effects on disease risk for unrelated infections and immune responses to vaccines. This study aimed to determine the immunomodulatory effects of BCG vaccination on immune responses to vaccines against SARS‐CoV‐2.

**Methods:**

Blood samples, from a subset of 275 SARS‐CoV‐2‐naïve healthcare workers randomised to BCG vaccination (BCG group) or no BCG vaccination (Control group) in the BRACE trial, were collected before and 28 days after the primary course (two doses) of ChAdOx1‐S (Oxford‐AstraZeneca) or BNT162b2 (Pfizer‐BioNTech) vaccination. SARS‐CoV‐2‐specific antibodies were measured using ELISA and multiplex bead array, whole blood cytokine responses to γ‐irradiated SARS‐CoV‐2 (iSARS) stimulation were measured by multiplex bead array, and SARS‐CoV‐2‐specific T‐cell responses were measured by activation‐induced marker and intracellular cytokine staining assays.

**Results:**

After randomisation (mean 11 months) but prior to COVID‐19 vaccination, the BCG group had lower cytokine responses to iSARS stimulation than the Control group. After two doses of ChAdOx1‐S, differences in iSARS‐induced cytokine responses between the BCG group and Control group were found for three cytokines (CTACK, TRAIL and VEGF). No differences were found between the groups after BNT162b2 vaccination. There were also no differences between the BCG and Control groups in COVID‐19 vaccine‐induced antigen‐specific antibody responses, T‐cell activation or T‐cell cytokine production.

**Conclusion:**

BCG vaccination induced a broad and persistent reduction in *ex vivo* cytokine responses to SARS‐CoV‐2. Following COVID‐19 vaccination, this effect was abrogated, and BCG vaccination did not influence adaptive immune responses to COVID‐19 vaccine antigens.

## Graphical abstract

Bacille Calmette–Guérin (BCG), the vaccine for tuberculosis, has immunomodulatory effects that may underpin its off‐target effects on infectious disease risk and vaccine responses. This study used blood samples taken before and 28 days after COVID‐19 vaccinations (2‐dose primary course), from healthcare workers randomised to receive BCG vaccine or no BCG, in the BRACE trial. We found that BCG vaccination caused long‐lasting reductions in immune responses to SARS‐CoV‐2. However, this suppressive effect of BCG was abrogated by COVID‐19 vaccinations.

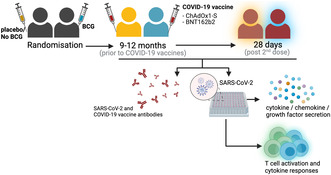

## Introduction

Bacille Calmette–Guérin (BCG) vaccine has been used for over 100 years to protect against tuberculosis (TB). In addition to protection against TB and other mycobacterial infections, BCG vaccination has off‐target (also known as ‘non‐specific’ or heterologous) immunological and clinical effects. These include protection from all‐cause mortality and infectious disease in infants in high‐mortality settings,^1^, ^2^, ^3^, ^4^, ^5^ as well as reduced incidence of infectious disease in the elderly.^6^, ^7^ Because of these beneficial off‐target effects of BCG vaccination, early in the COVID‐19 pandemic, BCG vaccination was trialled as a preventative measure to reduce the impact of COVID‐19 until specific vaccines could be developed. Findings from these trials have been inconsistent, although the larger trials did not show a protective effect of BCG vaccination.^8^ A major consideration in the interpretation of these trials and the potential off‐target effects of BCG vaccination are the possible effects of BCG vaccination on COVID‐19 vaccine immunity.

Prior or concurrent BCG vaccination can enhance antibody responses to some vaccines, including hepatitis B, polio, pneumococcus and influenza vaccines.^9^, ^10^, ^11^ BCG vaccination has also been shown to alter *ex‐vivo* vaccine‐specific cytokine and/or lymphocyte proliferation responses to DTP and hepatitis B vaccine,^10^, ^12^, ^13^ and reduce viremia to yellow fever vaccine.^14^ Preliminary studies in small cohorts reported that BCG re‐vaccination enhanced COVID‐19 vaccine responses.^15^, ^16^ However, subsequent larger studies found no effect of BCG vaccination (in the elderly) or BCG re‐vaccination (in healthcare workers) on antibody responses to COVID‐19 vaccines.^17^, ^18^

In the ‘BCG Vaccination to Reduce the Impact of COVID‐19 in Healthcare Workers’ (BRACE) randomised controlled trial (RCT), participants were randomised to BCG or no BCG (Control) to determine whether BCG vaccination reduces the incidence and severity of COVID‐19.^19^, ^20^ As COVID‐19 vaccines became available during the BRACE trial, we established the ‘BRACE COVID‐19‐specific vaccine’ (BCOS) sub‐study to determine the effect of prior BCG vaccination on immune responses to COVID‐19‐specific vaccinations.

## Results

**Figure 1:**
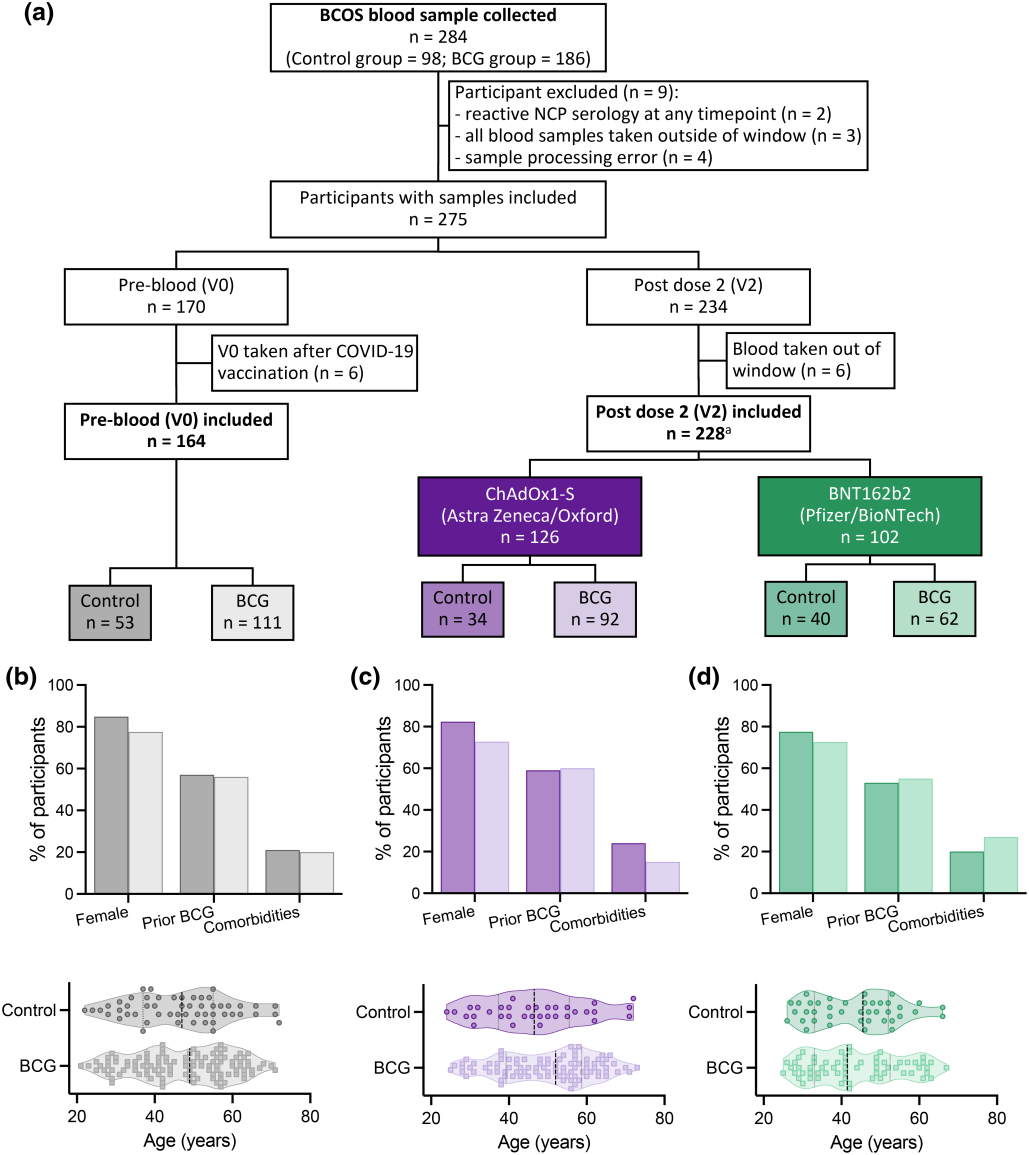
Participant flow diagram and demographics. **(a)** Consort diagram of samples included in analysis. ^a^124 with V0 samples. **(b–d)** Demographic data on participants with samples collected **(b)** prior to COVID‐19 vaccination (V0) and **(c, d)** 28 ± 3 days after the second dose (V2) of **(c)** ChAdOx1‐S vaccine or **(d)** BNT162b2 vaccine. BCG, bacille Calmette Guerin; BCOS, BRACE COVID‐19‐specific vaccine sub‐study; NCP, SARS‐CoV‐2 nucleocapsid.

Of 284 participants in Australia with blood samples collected for BCOS, 267 had samples eligible for inclusion. Of these, 164 participants had eligible pre‐vaccination (V0) samples and 228 had eligible V2 (28 ± 3 days post second dose) samples (Figure 1). The mean age of participants was 46.0 [standard deviation (SD) 12.3] years at randomisation in the BRACE trial with an older age among ChAdOx1‐S (Oxford‐AstraZeneca) recipients [mean 47.8 (SD 12.6) years] compared with BNT162b2 (Pfizer‐BioNTech) recipients [mean 43.7 (SD 11.6) years]. Participants received their first COVID‐19 vaccinations a mean of 11.2 (SD 1.5) months after randomisation in the BRACE trial. Participant age, prior BCG vaccination and comorbidities were similar between participants in the Control and BCG groups (Figure 1, Table 1). Participants were more likely to be female (78.5%), particularly among ChAdOx1‐S recipients in the Control group (Figure 1, Supplementary table 1).

**Table 1:** Participants with samples included in analysis

	Control group *n* = 94	BCG group *n* = 181
Age (years) at randomisation in the BRACE trial, mean (SD)	44.6 (12.1)	46.7 (12.4)
Sex		
Male	16 (17.0%)	42 (23.2%)
Female	78 (83.0%)	138 (76.2%)
Declined	0 (0.0%)	1 (0.6%)
COVID‐19 vaccine type		
ChAdOx1‐S	45 (47.9%)	109 (60.2%)
BNT162b2	48 (51.1%)	72 (39.8%)
Missing^a^	1 (1.1%)	0 (0.0%)
Months between randomisation and 1st COVID‐19 vaccination dose, mean (SD)	11.3 (1.5)	11.1 (1.5)
Days between 1st and 2nd COVID‐19 vaccination doses, mean (SD)	54.3 (32.8)	62.1 (32.5)
BCG‐vaccinated prior to BRACE trial		
Yes	50 (53.2%)	103 (56.9%)
No	44 (46.8%)	78 (43.1%)
Tuberculosis^b^ prior to BRACE trial		
Yes	5 (5.3%)	21 (11.6%)
No	77 (81.9%)	133 (73.5%)
Unsure	12 (12.8%)	27 (14.9%)
Any COVID‐19 comorbidities^c^		
Yes	18 (19.1%)	36 (19.9%)
Diabetes	2 (2.1%)	1 (0.6%)
Cardiovascular disease	7 (7.4%)	21 (11.6%)
Chronic respiratory disease	9 (9.6%)	16 (8.8%)
No	73 (77.7%)	144 (79.6%)
Missing	3 (3.2%)	1 (0.6%)
Obesity		
Yes	12 (12.8%)	24 (13.3%)
No	75 (79.8%)	142 (78.5%)
Missing	7 (7.4%)	15 (8.3%)
Smoking		
Yes	7 (7.4%)	8 (4.4%)
No	87 (92.6%)	173 (95.6%)
Occupation		
Allied Health	3 (3.2%)	2 (1.1%)
Clerical/Administrative duties	8 (8.5%)	17 (9.4%)
Doctor	16 (17.0%)	29 (16.0%)
Nurse/Midwife	39 (41.5%)	77 (42.5%)
Other role	28 (29.8%)	56 (30.9%)
Randomisation stage in BRACE trial		
Stage 1	78 (83.0%)	136 (75.1%)
Stage 2	16 (17.0%)	45 (24.9%)
Scar development after BCG vaccination in BRACE trial		
Yes	—	130 (71.8%)
No	—	48 (26.5%)
Any other vaccinations between randomisation and blood collection		
V0	3 (3.2%)	6 (3.3%)
V1	2 (2.1%)	10 (5.5%)
V2	13 (13.8%)	29 (16.0%)

^a^ V0 (pre‐COVID‐19 vaccination) sample only.

^b^ Positive tuberculin‐skin‐test or treatment for tuberculosis ever prior to randomisation in BRACE trial.

^c^ At BRACE trial randomisation: diabetes (any type), cardiovascular disease (including hypertension) or chronic respiratory disease (including asthma and chronic obstructive pulmonary disease).

**Figure 2:**
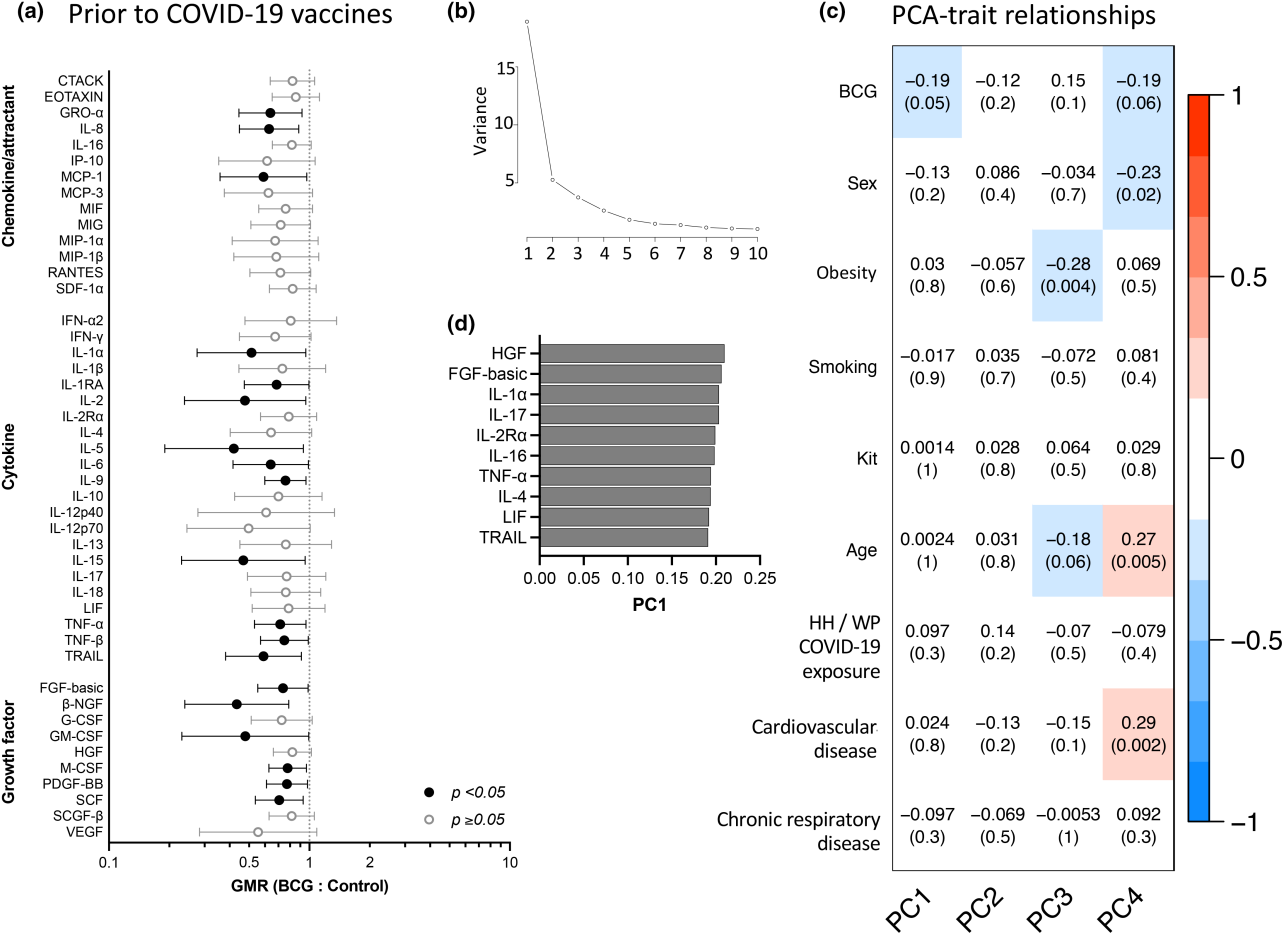
BCG vaccination reduces SARS‐CoV‐2 cytokine responses prior to COVID‐19 vaccination. Whole blood cytokine responses to γ‐irradiated SARS‐CoV‐2 (iSARS) in blood samples taken before COVID‐19 vaccinations (V0). **(a)** Forest plots depicting adjusted geometric mean ratios (GMR) and 95% confidence intervals for the effect of BCG vaccination determined by multivariable linear regression (Supplementary table 2). GMR > 1.0 indicates responses that were higher for BCG‐vaccinated (*n* = 70) compared to Control (*n* = 35) participants. *P*‐values < 0.05 are depicted in black. **(b–d)** Unsupervised principal component analysis (PCA) of *Z*‐scaled iSARS cytokine responses with COMBAT correction for cytokine assay kit. **(b)** Scree plot of eigenvalues for the first 10 principal components. **(c)** Heatmap of coefficient of determination (and associated *P*‐values) for influencing factors for the first 4 PCs. **(d)** Contribution (loading) for top 10 cytokines contributing to difference in PC1. BCG, bacille Calmette Guérin; HH, household; PC, principal component; WP, workplace.

To determine whether BCG vaccination administered almost a year earlier influenced immune responses to SARS‐CoV‐2, cytokine responses to stimulation with γ‐irradiated SARS‐CoV‐2 (iSARS) were compared between the BCG and Control groups in samples taken prior to COVID‐19 vaccination (V0). Participants had V0 blood taken at 6.6 to 13.9 months after randomisation (Control group mean 11.0 (SD 1.7) months; BCG group mean 10.8 (SD 1.7) months). Following *ex vivo* stimulation of whole blood samples collected prior to COVID‐19 vaccination with iSARS, all but two of the 48 cytokines assessed were lower in the BCG group than in the Control group. This was statistically significant for 19 cytokines: basic FGF *P* = 0.040; GM‐CSF *P* = 0.048; GRO‐α *P* = 0.016; IL‐1α *P* = 0.037; IL‐1Ra *P* = 0.046; IL‐2 *P* = 0.038; IL‐5 *P* = 0.033; IL‐6 *P* = 0.044; IL‐8 *P* = 0.008; IL‐9 *P* = 0.023; IL‐15 *P* = 0.036; MCP‐1 *P* = 0.038; M‐CSF *P* = 0.024; β‐NGF *P* = 0.007; PDGF‐BB *P* = 0.033; SCF *P* = 0.014; TNF‐α *P* = 0.027; lymphotoxin‐α (TNF‐β) *P* = 0.039; TRAIL *P* = 0.018 (Figure 2a, Supplementary figure 1, Supplementary tables 2 and 3a). Consistent with a finding of lower cytokine response to iSARS 11 months after randomisation, analysis of samples taken three months after randomisation also showed reduced responses in the BCG group compared with the Control group (Supplementary figure 2). However, at three months post randomisation, this reduced response to iSARS was only statistically significant for GRO‐α *P* = 0.048, IL‐2 *P* = 0.032 and IL‐5 *P* = 0.005.

Consistent with the documented waning of BCG vaccination‐induced CD4^+^ T‐cell IFN‐γ responses to mycobacteria over 6–12 months,^21^, ^22^ analysis of cytokine responses to *ex vivo* BCG stimulation at V0 (11 months post randomisation) showed similar cytokine responses in the BCG and Control groups, including for IFN‐γ (Supplementary table 2). Similarly, cytokine responses to iVero, heat‐killed (HK) *Candida albicans*, HK *Escherichia coli*, HK *Staphylococcus aureus* and R848 were similar between the BCG and Control groups (Supplementary tables 2 and 3a).

There is no accepted immunological correlate of protection for BCG vaccination. However, failure to develop a scar after BCG vaccination is often considered to represent ineffective vaccination^23^, ^24^ and the presence of BCG scar, used as a surrogate indicator of vaccination status, has been associated with beneficial off‐target effects.^25^, ^26^ Therefore, we did a sensitivity analysis excluding participants in the BCG group who did not develop a BCG vaccination scar by 12 months post randomisation. In the sensitivity analysis, the lower cytokine responses to iSARS in the BCG group compared with the Control group was retained; however, because of lower participant numbers, there was decreased statistical power in the sensitivity analysis than for the as‐treated analysis (Supplementary table 2). For responses to BCG stimulation, there was a small difference in LIF responses between the BCG group (which included only those who developed a scar) and the Control group (Supplementary table 2). All other cytokine responses to BCG stimulation remained similar between the BCG and Control group in the sensitivity analysis. There were also only minor differences in the effects of BCG vaccination in the sensitivity analysis for unrelated stimuli, the most notable being for stimulation with *E. coli* and R848, for which four additional cytokines (*E. coli*: FGF‐basic, IL‐6, IL‐17 and LIF; R848: FGF‐basic, IL‐2Rα, IL‐17 and LIF) had significantly lower cytokine responses in the BCG group than in the Control group (Supplementary table 2).

Principal component analysis (PCA) of all detected cytokines confirmed the differences in iSARS responses observed between the BCG and Control groups prior to COVID‐19 vaccination (V0). BCG vaccination had the strongest influence on cytokine responses (observable in PC1), while sex, obesity, age and cardiovascular disease contributed to a lesser degree (Figure 2b and c). Cytokines that mediated the influence of BCG on cytokine responses to stimulation with iSARS are shown in Figure 2d, and include HGF, IL‐1α, IL‐17 and TRAIL.

**Figure 3:**
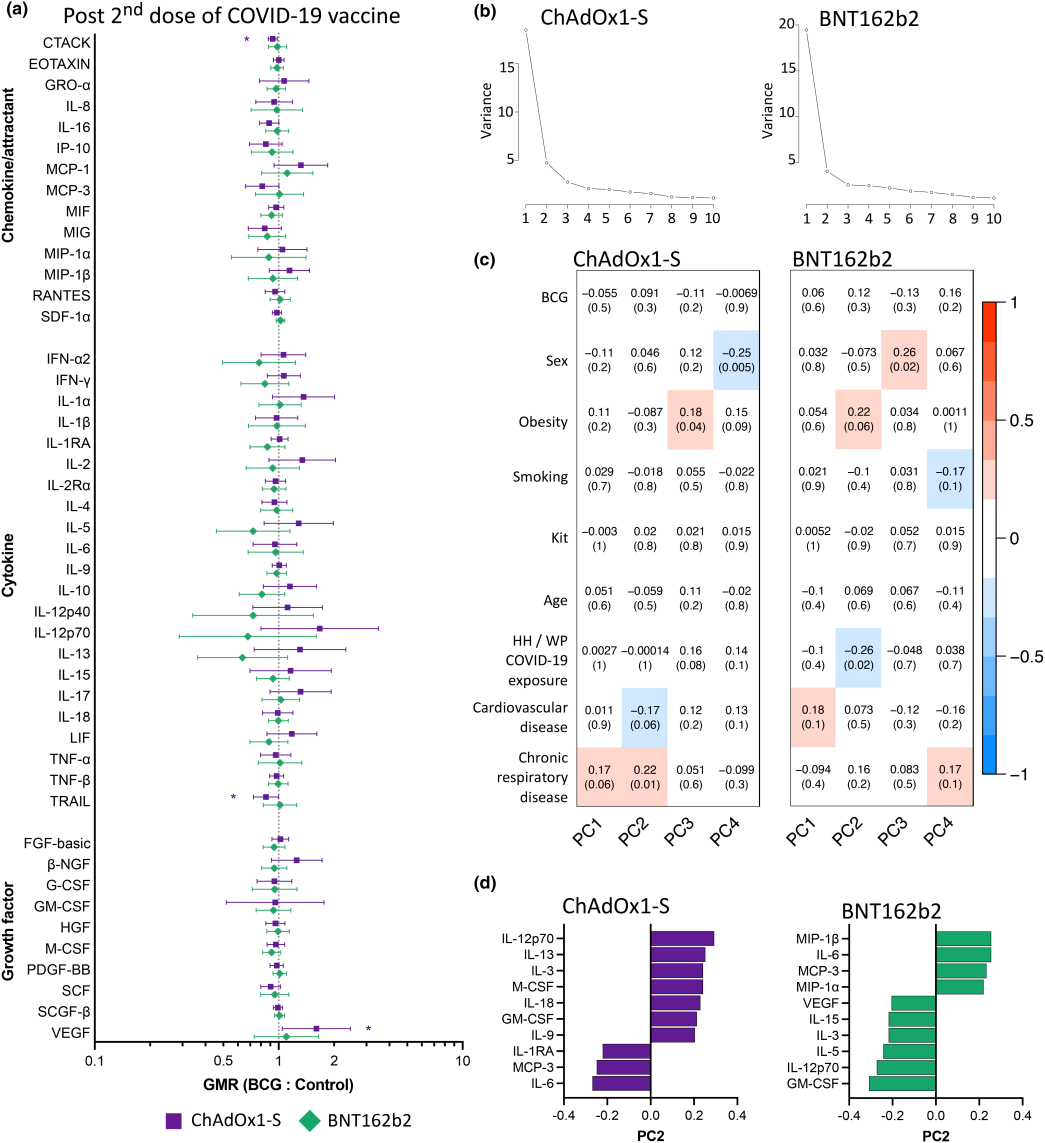
BCG vaccination does not alter SARS‐CoV‐2 cytokine responses following COVID‐19 vaccinations. Whole blood cytokine responses to γ‐irradiated SARS‐CoV‐2 (iSARS) in blood samples taken 28 days after the second dose of ChAdOx1‐S (purple; BCG group *n* = 85, Control group *n* = 33) or BNT162b2 (green; BCG group *n* = 40, Control group *n* = 26) vaccination (V2). **(a)** Forest plots depicting adjusted geometric mean ratios (GMR) and 95% confidence intervals for the effect of BCG vaccination determined by multivariable linear regression. GMR > 1.0 indicates responses that were higher for BCG‐vaccinated compared with Control participants. *P < 0.05. **(b–d)** Unsupervised principal component analysis (PCA) of iSARS‐induced cytokine responses 28 days after the second dose of ChAdOx1‐S (left) or BNT162b2 (right) vaccination. Prior to PCA, cytokine concentrations were *Z*‐scaled and PCA was corrected for cytokine assay kit using COMBAT. **(b)** Scree plot of eigenvalues for the first 10 principal components. **(c)** Heatmap of coefficient of determination (and associated *P*‐values) for influencing factors for the first 4 PCs. **(d)** Contribution (loading) for top 10 cytokines contributing to difference in PC2. BCG, bacille Calmette Guérin; HH, household; PC, principal component; WP, workplace.

Given the strong and persistent effect of BCG vaccination on *ex vivo* cytokine responses to iSARS, we assessed whether prior BCG vaccination altered iSARS cytokine responses administration of a COVID‐19 adenoviral vectored (ChAdOx1‐S) or mRNA (BNT162b2) vaccine. Twenty‐eight days after the second dose of ChAdOx1‐S, differences in cytokine responses to iSARS between the BCG and Control group could no longer be detected for most cytokines with the exception of a significantly lower response in the BCG group for CTACK (geometric mean ratio (GMR) 0.93, 95% confidence interval (95% CI) 0.88–0.98; *P* = 0.009) and TRAIL (GMR 0.85, 95% CI 0.73–1.0; *P* = 0.046) and higher responses for VEGF (GMR 1.60, 95% CI 1.05–2.46; *P* = 0.030) (Figure 3a, Supplementary tables 3b and 5). The effect of BCG vaccination on VEGF responses was also evident 28 days after the first dose (V1) of ChAdOx1‐S (GMR 2.07, 95% CI 3.82 to 1.13; *P* = 0.020; Supplementary figure 2). No other significant effects of BCG vaccination on cytokine responses to iSARS were observed at V1. Among BNT162b2 recipients, there was no evidence of a difference in iSARS cytokine responses between participants in the BCG and Control groups (Figure 3a, Supplementary figure 1, Supplementary tables 3b and 5). For several cytokines, the effects of BCG vaccination on cytokine response to iSARS appeared to be different between ChAdOx1‐S and BNT162b2 recipients (Supplementary figure 2). However, differences in the age of ChAdOx1‐S compared with BNT162b2 recipients and differences in the intervals between primary and secondary doses because of vaccination policy precluded direct comparison of the effects of BCG vaccination in these two groups. For both ChAdOx1‐S and BNT162b2 recipients, sensitivity analysis excluding participants in the BCG group who did not develop a BCG vaccination scar by 12 months post randomisation showed similar effects (Supplementary table 6).

Analysis of cytokine response by PCA also found that BCG vaccination in the BRACE trial was not a major contributor to variability in cytokine responses to iSARS following two doses of ChAdOx1‐S or BNT162b2 (Figure 3b and c). For ChAdOx1‐S recipients, the demographic factors with the greatest influence on the variability in cytokine responses included chronic respiratory disease and cardiovascular disease, with IL‐12p70 and IL‐6 the largest contributors, as well as sex and obesity (Figure 3c and d). For BNT162b2 recipients, the demographic factors with greatest influence on the variability in cytokine responses included obesity and workplace/household COVID‐19 exposure, with GM‐CSF and IL‐12p70 the largest contributors, as well as sex (Figure 3c and d).

**Figure 4:**
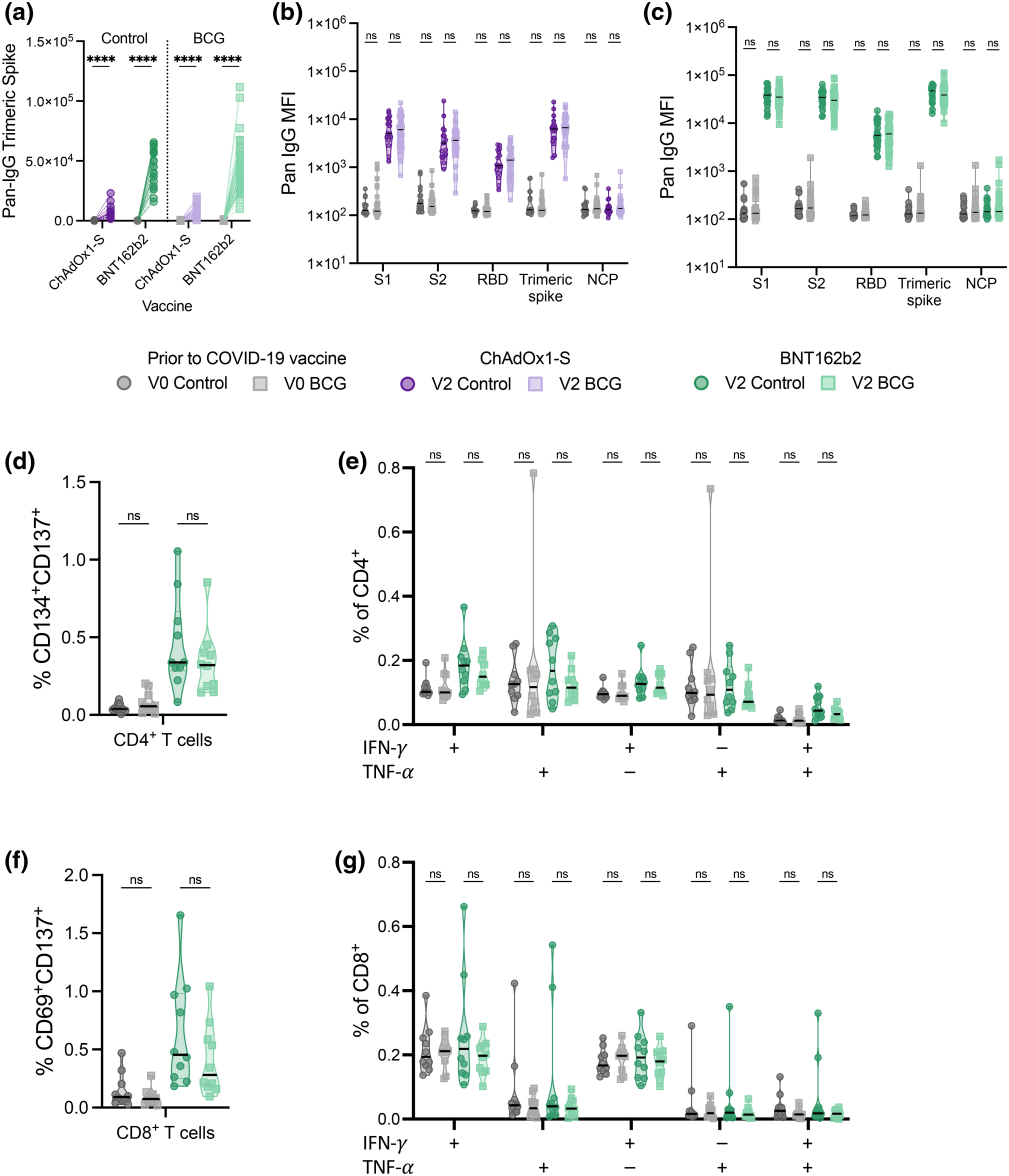
BCG vaccination does not alter SARS‐CoV‐2 specific antibody or T‐cell responses to COVID‐19 vaccinations. **(a)** Dotplot presenting the change in anti‐SARS‐CoV‐2 trimeric Spike Pan‐IgG MFI before (grey) and 28 days after the second ChAdOx1‐S (purple, *n* = 64) or BNT162b2 (green, *n* = 60) dose in BCG vaccinated and Control participants. Differences before and after COVID‐19 vaccinations were determined by Wilcoxon signed‐rank test ****P < 0.0001. **(b, c)** Violin graph with scatter dot‐plot presenting anti‐Spike (S1, S2, RBD and trimeric Spike) and anti‐nucleocapsid (NCP) Pan‐IgG MFI. Differences between BCG‐vaccinated (ChAdOx1‐S *n* = 47, BNT162b2 = 38) and Control (ChAdOx1‐S *n* = 17, BNT162b2 *n* = 22) participants were determined by Wilcoxon rank‐sum test. **(d–g)** Violin graph with scatter dot‐plot presenting percentage of **(d,f)** activated and **(e, g)** cytokine producing **(d, e)** CD4^+^ and **(f, g)** CD8^+^ T cells. Differences between BCG‐vaccinated (*n* = 10) and Control (*n* = 10) participants were determined by bootstrapped quantile regression. BCG, bacille Calmette Guérin; NCP, nucleocapsid; ns, non‐significant (*P* ≥ 0.05); RBD receptor binding domain; S1, subunit 1; S2, subunit 2; V0, before COVID‐19 vaccination; V2, 28 days after COVID‐19 vaccination.

While BCG‐induced trained innate immunity is largely reported in innate immune cells, studies suggest that BCG vaccination may alter antibody responses to subsequent vaccines. We therefore used a multiplex bead assay to assess whether BCG vaccination altered the level or type of antibody response induced following ChAdOx1‐S and BNT162b2 vaccination in a subset of participants with paired pre‐(V0) and post‐(V2) vaccination samples. Participants in both the BCG and Control groups had robust anti‐Spike (S1, S2, RBD and trimeric Spike) IgG responses following two doses of COVID‐19 vaccinations (Figure 4a, Supplementary table 7). There were no differences between the BCG and Control group in anti‐Spike (S1, S2, receptor binding domain (RBD) and trimeric Spike) IgG1, IgG2, IgG3, IgG4, pan IgG or IgA responses before or 28 days after two doses of ChAdOx1‐S or BNT162b2 vaccinations (Figure 4b and c, Supplementary table 8). A validation analysis using ELISA to determine anti‐RBD and anti‐Spike IgG responses in all participants also showed no difference between the BCG and Control groups before or 28 days after two doses of COVID‐19 vaccinations (Supplementary figure 3).

There is limited evidence for direct effects of BGC vaccination on off‐target T‐cell responses.^27^ However, BCG vaccination can induce changes in T‐cell cytokine responses to unrelated vaccines.^13^ We therefore assessed whether BCG vaccination altered T‐cell responses following COVID‐19 vaccination. There was robust activation of CD4^+^, CD4^+^ T follicular and CD8^+^ T cells in response to Spike antigen stimulation in peripheral blood mononuclear cells (PBMCs) from both the BCG and Control groups following two doses of BNT162b2 vaccination (Supplementary figure 4, Supplementary table 8). Spike stimulation induced CD4^+^, CD4^+^ T follicular or CD8^+^ T‐cell activation was similar between participants in the BCG and Control groups before and after BNT162b2 vaccination (Figure 4d and f, Supplementary figure 5, Supplementary table 9). There was a statistically significant (*P* < 0.05) increase in Spike‐specific IFN‐γ and TNF‐α‐producing CD4^+^ T cells (IFN‐γ‐, TNF‐α‐ and IFN‐γ/TNF‐α‐producing) following two doses of BNT162b2 vaccination in the BCG group but not the Control group (Supplementary figure 4, Supplementary table 8). However, when directly comparing the cytokine responses post vaccination, there were no statistically significant differences between the two groups in IFN‐γ or TNF‐α producing CD4^+^ or CD8^+^ T cells (Figure 4e and g, and Supplementary table 9).

## Discussion

In this sub‐study of healthcare workers in the BRACE trial, BCG vaccination reduced *ex vivo* cytokine responses to SARS‐CoV‐2 almost a year after randomisation, but there was limited evidence for an effect on SARS‐CoV‐2‐specific cytokine, antibody or T‐cell responses following ChAdOx1‐S or BNT162b2 COVID‐19 vaccinations.

The protective effects of BCG vaccination against unrelated infectious diseases, together with the observed off‐target effects on immune responses to unrelated pathogens, made BCG vaccination a promising intervention for reducing the impact of the novel pathogen SARS‐CoV‐2 until specific vaccines could be developed.^28^, ^29^ Multiple trials were embarked upon to investigate whether BCG vaccination could protect against and reduce the severity of COVID‐19.^30^ One concern at the outset of BCG vaccination trials was that, by increasing immune responses to SARS‐CoV‐2, BCG vaccination may exacerbate COVID‐19 symptoms.^28^ Although the majority of trials were underpowered, the largest among them (each with > 2000 participants) found an increase in symptomatic COVID‐19 among BCG‐vaccinated participants.^8^, ^20^, ^31^, ^32^ However, this increased risk of symptomatic COVID‐19 was only statistically significant in the BRACE trial (*n* = 3988), the international RCT from which participants in this immunological sub‐study were sampled.^32^ The increased risk of COVID‐19 in BCG‐vaccinated participants paired with reduced cytokine responses to iSARS suggests that, rather than inducing a pro‐inflammatory state, BCG vaccination could have dampened early immune responses to SARS‐CoV‐2, thus delaying pathogen clearance and leading to more symptomatic disease.

Studies of the immunomodulatory effects of BCG vaccination in adults have typically found that BCG increases cytokine responses following *ex vivo* stimulation with unrelated (i.e. non‐mycobacterial) microbial ligands. However, studies of BCG immunomodulatory effects in humans have largely focussed on bacterial and fungal pathogens, and Toll‐like Receptor agonists.^33^, ^34^, ^35^, ^36^, ^37^ In a human challenge model of infection using the live‐attenuated yellow fever virus vaccine, BCG vaccination induced trained innate immunity and reduced viremia, but had no effect on the *in vitro* IFN‐γ response to yellow fever virus.^14^ For influenza virus, a small (*n* = 40) RCT of BCG vaccination in adults found no effect of BCG vaccination on *ex vivo* responses to live influenza virus 2 weeks after randomisation,^11^ but a recent trial among adults, aged 60 years or above, found higher *in vitro* IL‐6, IL‐1β and TNF‐α responses to heat‐inactivated influenza virus for BCG‐vaccinated compared with placebo‐vaccinated participants 12 months after randomisation.^38^ The trial of older adults also found higher IL‐6 responses to heat‐inactivated SARS‐CoV‐2 for BCG‐vaccinated participants but no effect on the other cytokines tested.^38^ Notably, however, there was no difference between BCG‐vaccinated and placebo‐vaccinated participants in SARS‐CoV‐2 infection in that study. In contrast to these studies, our previous findings comparing samples before and three months after randomisation,^39^ and our current study, show that BCG vaccination induces a broad and persistent reduction in whole blood cytokine responses to *ex vivo* stimulation with iSARS. This suppressive effect was evident three months after randomisation but stronger at 11 months. The reason for the enhanced effect over time is unclear; however, studies of BCG off‐target effects on bacterial and fungal responses also show increasing effects at one year compared with three months after vaccination.^34^ There are a number of possible explanations for the differences in the reported effects of BCG vaccination on responses to viral ligands. These include participant characteristics, such as participant age and ethnicity, which have been shown to influence the effect of BCG vaccination on COVID‐19^20^ and immune responses to SARS‐CoV‐2, respectively.^40^ Other contributory factors might include differences in study design, in particular the use of whole blood vs PBMCs, and heat vs γ‐irradiation inactivated SARS‐CoV‐2. Because of differences in study design, it is unclear whether the effect observed in our study is specific to SARS‐CoV‐2 or more generalised across response to other viruses. Analysis of responses to R848, an agonist of TLR7/8 that recognise viral single‐stranded RNA, showed only minor effects in this study, primarily in the sensitivity analysis excluding participants in the BCG group without a scar. This minor difference between the intervention groups suggests that the effect of BCG vaccination is not broadly conserved across single‐stranded RNA viruses and likely functions downstream of alternative pattern recognition receptors.

In addition to effects on unrelated pathogens, BCG vaccination may also alter responses to subsequent vaccinations. Early studies suggested that BCG vaccination enhanced humoral and *in vitro* cytokine responses to vaccine antigens.^27^, ^41^ However, recent infant and adult studies found only non‐statistically significant effects of BCG vaccination on subsequent antibody responses to routine vaccines.^14^, ^42^ Overall, studies reporting the effect of BCG vaccination on adaptive immune responses induced by other vaccines have shown inconsistent effects.^14^, ^27^, ^41^, ^42^ For COVID‐19 vaccines, studies in mice have reported that BCG vaccination increases adaptive immune responses to SARS‐CoV‐2 Spike vaccines and that prior or co‐administration reduces severity of SARS‐CoV‐2 infection.^43^, ^44^ In two large immunological studies of participants in RCTs, one among the elderly in the Netherlands (*n* = 945) and the other among healthcare workers in Brazil (*n* = 874), there was no difference between BCG‐vaccinated and placebo‐vaccinated participants in IgG responses to COVID‐19 vaccines (ChAdOx1‐S and BNT162b2 in the Netherlands, median of 347 days after randomisation; ChAdOx1‐S and CoronaVac in Brazil mean of 80 days after randomisation).^17^, ^18^ Our finding of no effect of BCG vaccination on vaccine‐specific antibody responses following ChAdOx1‐S and BNT162b2 vaccination is consistent with these studies. In contrast, one small observational study (*n* = 37) of healthcare workers in India found that BCG re‐vaccination enhanced antibody and Spike‐specific T‐cell cytokine responses to the adenovirus‐based ChAdOx1‐S vaccine.^15^ However, these effects varied between high and low Spike‐antigen responders and was potentially prone to type 1 error having included less than five participants in the vaccination group. A second small study in Mexico (*n* = 60) found that, compared with placebo‐vaccination, BCG re‐vaccination of healthcare workers enhanced neutralising antibody responses to BNT162b2.^16^ A difference between our study and these earlier studies in India and Mexico is the interval between BCG and COVID‐19 vaccinations: 11 months in the present study, compared with 30 days in the others. In our study, the limited differences in cytokine responses to iSARS and the absence of an observed difference in antibody responses following the COVID‐19 vaccination together with the lack of an observed difference in T‐cell responses between BCG‐vaccinated and placebo‐vaccinated BNT162b2 recipients suggest that recent BCG vaccination (11 months prior) does not impact immune responses to COVID‐19 vaccinations.

The strengths of our study include the large number of participants from a RCT of BCG vaccination and the inclusion of only SARS‐CoV‐2‐naïve participants. As part of the BRACE trial, these participants had weekly prospective follow‐up and 3‐monthly anti‐NCP antibody testing, enabling the exclusion of any participants with prior SARS‐CoV‐2 infection. This ensured that observed effects of BCG before and after COVID‐19 vaccinations were not confounded by adaptive immune responses to prior SARS‐CoV‐2 infection. An additional strength is the analysis of a range of responses to COVID‐19 vaccines to provide a comprehensive analysis of the effect of BCG. A further strength was the analysis of the effect of BCG vaccination on response to COVID‐19 vaccines based on two distinct platform technologies. This enabled us to ascertain that the limited effect of BCG after COVID‐19 vaccination was not vaccine/platform specific and likely related to the strength of the peak (four weeks after a dose) COVID‐19‐specific response largely overcoming any effects of BCG.

A limitation of our study was the inability to determine whether BCG vaccination was effective in all participants in the BCG group. There is no correlate of protection for BCG vaccination and therefore no measure to determine effectiveness of vaccination. Canonically, BCG vaccination increases *in vitro* IFN‐γ responses to BCG and mycobacterium tuberculosis through antigen‐specific CD4^+^ T cells.^45^, ^46^ This response peaks 10–12 weeks after vaccination and wanes over 6–12 months, the period of time that responses were measured in our participants.^21^, ^22^ However, BCG vaccination induced IFN‐γ responses to mycobacterial stimulation are not consistently observed across and within populations,^47^, ^48^, ^49^, ^50^, ^51^ and are not correlated with either protection against tuberculosis^49^, ^52^, ^53^ or induction of trained immunity responses.^14^, ^54^ Therefore, the absence of a difference in IFN‐γ responses between BCG vaccinated and Control participants in our study is not indicative of ineffective BCG vaccination. An alternative measure of effective BCG vaccination is the development of a keloid scar at the injection site. While BCG scar size is not correlated with protection against TB,^55^ the absence of a BCG scar is often used as a surrogate for ‘no BCG vaccination’ in cohort studies of specific and off‐target effects and has been used as an indicator for BCG re‐vaccination.^25^, ^26^, ^56^, ^57^, ^58^ In our study, sensitivity analysis including only BCG‐vaccinated participants who developed a scar by 12 months post vaccination showed no overall difference in the observed effect of BCG vaccination on responses to BCG or SARS‐CoV‐2 indicating that the lack of observed difference after COVID‐19 vaccination was not because of failed BCG vaccination.

A further limitation of this study includes the receipt of influenza vaccination on the day of randomisation for participants recruited in Stage 1 of the BRACE trial. The clinical off‐target effects of BCG vaccination are proposed to be retained with co‐vaccination^5^; however, it is possible that the influenza vaccination in the Stage 1 participants influenced the observed effect of BCG.^59^, ^60^ To account for any effect of influenza co‐vaccination, we adjusted for BRACE trial stage in the regression analyses. An additional limitation is the unequal recruitment of BCG‐vaccinated and Control participants. Although participants were randomised 1:1 to the BCG vaccination or Control group in the BRACE trial, participants who did not receive BCG vaccination (which might be assumed by participants because of lack of BCG scar formation months after randomisation) were less likely to enrol in the sub‐study. Finally, differences in participant age and the intervals between primary and secondary doses (12 weeks for ChAdOx1‐S and three weeks for BNT162b2), dictated by national vaccination policy during the study, limited the ability to compare the off‐target effects of BCG vaccination between the two vaccine platforms. This difference in age is particularly relevant as the age at which BCG vaccination is administered may influence its off‐target effects against SARS‐CoV‐2.^8^, ^32^

In SARS‐CoV‐2‐naïve healthcare workers in Australia, BCG vaccination induced a broad and long‐lasting reduction in *ex vivo* cytokine responses to SARS‐CoV‐2. This effect was overcome by ChAdOx1‐S or BNT162b2 vaccination, after which there was limited observed effect of BCG vaccination on immune responses to SARS‐CoV‐2 or COVID‐19 vaccine antigens.

## Methods

### Study setting

This study is an exploratory sub‐study of the BRACE trial (NCT04327206), an international RCT that took place from March 2020 to April 2022.

### BRACE trial

The protocol for the BRACE trial is available at clincaltrials.gov.^61^ Briefly, healthcare workers were recruited to the BRACE trial in two stages. In Stage 1 (recruitment in Australia from March 2020 to May 2020), participants were randomised 1:1 to receive BCG vaccination (BCG group) or no BCG (Control group) on the day of their seasonal influenza vaccination. In Stage 2 (recruitment in Australia, Spain, the Netherlands, the United Kingdom and Brazil from May 2020 to April 2021), participants were randomised 1:1 to receive BCG vaccination (BCG group) or saline placebo (Control group). Participants randomised to the BCG group in both stages received a single 0.1 mL intradermal dose of BCG‐Denmark vaccine (Danish strain 1331 AJ Vaccines, Copenhagen, Denmark). Participants were followed up for 12 months following randomisation with weekly symptom reporting (including COVID‐19 test results and daily symptom recording for episodes of illness) and 3‐monthly detailed surveys. Peripheral blood collected at randomisation and 3‐monthly throughout the trial was tested for antibodies against SARS‐CoV‐2 NCP protein to identify SARS‐CoV‐2 infections prior to and during the BRACE trial.^20^

### Randomisation and blinding

Randomisation in the BRACE trial was done using a web‐based procedure (REDCap®),^62^ in randomly permuted blocks of variable length. Randomisation was stratified by stage, site, age group and presence of COVID‐19 comorbidity. Investigators and trial staff (excluding those involved in randomisation and safety follow‐up/reporting) were blinded to the randomisation group throughout the trial.

### BRACE COVID‐19‐specific vaccine sub‐study

This sub‐study included BRACE trial participants recruited to the BCOS sub‐study from BRACE trial study sites in Australia (Victoria and South Australia). Participants were eligible for inclusion in BCOS if they had consented to be contacted for future ethically approved projects and were recruited to the BRACE trial at a study site participating in BCOS. Participants were excluded from eligibility in BCOS if they expected to be unable to provide a blood sample 28 days after the first and second dose of a COVID‐19 vaccination, or if they had a positive SARS‐CoV‐2 test at any time prior to recruitment to BCOS.

Study visits for blood collection were prior to the first dose of a COVID‐19 vaccine (Visit 0, V0), 28 (±3) days after the first dose (V1) and 28 (±3) days after the second dose (V2). The recommended three‐week interval between the first and second dose of BNT162b2 at the time of this study precluded inclusion of a V1 study visit 28 days after the first dose for BNT162b2 recipients.

### COVID‐19 vaccination

Participants received their COVID‐19 vaccinations through the Australian COVID‐19 vaccination programme. At the time of the BCOS, the ChAdOx1‐S or BNT162b2 vaccines were administered in a two‐dose prime‐boost schedule in Australia with recommended intervals of 12 and three weeks, respectively.

### Sample collection

Peripheral blood samples were collected between 2 March 2021 and 24 September 2021. Peripheral blood was collected in CAT Serum Separator Clot Activator tubes (Greiner Bio‐One, Kremsmünster, Austria) and sodium heparin tubes (BD Biosciences, Franklin Lakes, USA).

### Serology testing

Peripheral blood collected in CAT tubes was centrifuged and serum was stored at −80°C until testing. Where a V0 serum sample was not available, the participant's most recent plasma sample available prior to COVID‐19 vaccination (from BRACE trial 3‐monthly samples) was used (*n* = 8). For prior SARS‐CoV‐2 infection testing, anti‐SARS‐CoV‐2 NCP total antibodies were measured using the Cobas Elecsys Anti‐SARS‐CoV‐2 assay as per the manufacturer's instructions (Roche, Basel, Switzerland).^63^

Anti‐SARS‐CoV‐2 Spike and anti‐SARS‐CoV‐2 receptor binding domain (RBD) IgG in serum and plasma samples were quantified by ELISA as previously described.^64^, ^65^

### Multiplex antibody analysis

To characterise antigen‐specific serum antibody responses, we utilised a panel of SARS‐CoV‐2 antigen‐coupled beads in a custom multiplex array, as previously described.^66^, ^67^ Briefly, magnetic carboxylated multiplex beads (Bio‐Rad, Hercules, USA) were coupled with 100 μg antigen [SARS‐CoV‐2 Trimeric Spike, Spike 1 (S1), Spike 2 (S2) (Sino Biological, Beijing, China)] per 1.25 × 10^7^ beads or 49.7 μg antigen [SARS‐CoV‐2 RBD (gift from Adam Wheatley)] per 1.25 × 10^7^ beads. 1000 beads/bead region diluted in PBS containing 0.1% bovine serum albumin (BSA) (incubation buffer) were added to each well (in a total of 25 μL volume per well) in black, clear‐bottom 384‐well plates (Greiner Bio‐One; 781 906). A volume of 25 μL serum diluted in PBS was added to each well (1:3200 for *Pan*‐IgG and IgG1; 1:800 for IgG2‐4 and IgA1) and plates were incubated on a plate shaker overnight at 4°C. Wells were then washed with PBS 0.05% Tween‐20 (PBST) and PE‐conjugated mouse anti‐human isotype antibodies (*Pan*‐IgG: Southern Biotech, Birmingham, USA, 9040–09, clone JDC‐10; IgG1: Southern Biotech, 9052–09, clone 4E3; IgG2: Southern Biotech, 9070–09, clone HP6002; IgG3: Southern Biotech, 9210–09, clone HP6050; IgG4: Southern Biotech, 9200–09, clone HP6025; IgA1: Southern Biotech, 9130–09, clone B3506B4) were diluted to 1.3 μg mL^−1^ and added at 25 μL volume per well before incubation for 2 h at room temperature on a plate shaker. Plates were then washed with PBST, and beads were resuspended in 50 μL of sheath fluid per well. The level of PE signal associated with each bead region in each well, reported as median fluorescence intensity (MFI), was determined by a FLEXMAP 3D Luminex instrument system (Luminex, Austin, USA). Double background subtraction was performed by first subtracting background measured in blank (buffer only) wells and subtracting background signal measured for BSA blocked control beads in each well. Haemagglutinin H1 (A/California/07/2009; H1 (Cal/09); Sino Biological) coupled beads were included as the positive control.

### Whole blood stimulation

Peripheral blood collected in sodium heparin tubes was diluted 1:1 with RPMI 1640 medium containing GlutaMAX Supplement and HEPES [Gibco, Life Technologies (Thermo Fisher Scientific), Waltham, USA] and added to pre‐prepared stimulation assay strips as described previously.^36^, ^39^ The final concentrations of stimuli in the assay strips were as follows: 1:9 γ‐irradiated mock‐infected Vero‐cell supernatants (iVero) and 1:9 γ‐irradiated SARS‐CoV‐2 [hCoV‐19/Australia/VIC01/2020 (VIC01, NCBI: MT007544.1)] infected Vero‐cell supernatants (iSARS) 10^6.2^ TCID_50_ mL^−1^, BCG‐Denmark (Serum Statens Institut, Copenhagen, Denmark) 75 μg mL^−1^, HK *C. albicans* 1.0 × 10^6^ CFU mL^−1^, HK *E. coli* 1.0 × 10^6^ CFU mL^−1^, HK *S. aureus* 1.0 × 10^7^ CFU mL^−1^ (HK pathogens were clinical isolates from children with invasive disease at the Royal Children's Hospital Melbourne^36^) and resiquimod (R848, InvivoGen, San Diego, USA) 3.5 μg mL^−1^. Whole blood was stimulated at 37°C (5% CO_2_) for 20 (±2) hours.^36^ Following stimulation, supernatants were harvested and stored at −80°C for future cytokine analysis.

### Cytokine analysis

Supernatants from stimulated whole blood were diluted 1:4 prior to quantification of secreted cytokines, chemokines and growth factors using the Bio‐Plex Pro Human Cytokine 48‐Plex Screening Panel (Bio‐Rad) according to the manufacturer's instructions. The following analytes were measured: CTACK, eotaxin, basic FGF, G‐CSF, GM‐CSF, GRO‐α, HGF, IFN‐α2, IFN‐γ, IL‐1α, IL‐1β, IL‐1Ra, IL‐2, IL‐2rα, IL‐3, IL‐4, IL‐5, IL‐6, IL‐7, IL‐8, IL‐9, IL‐10, IL‐12p40, IL‐12p70, IL‐13, IL‐15, IL‐16, IL‐17, IL‐18, IP‐10, LIF, MCP‐1, MCP‐3, M‐CSF, MIF, MIG, MIP‐1α, MIP‐1β, β‐NGF, PDGF‐BB, RANTES, SCF, SCGF‐β, SDF‐1α, TNF‐α, TNF‐β, TRAIL and VEGF. Data were acquired on the Bio‐Plex 200 system using the BioPlex Manager™ 6.1 Software (Bio‐Rad).

### T‐cell stimulation

PBMCs were isolated from peripheral blood collected in sodium heparin tubes by density centrifugation using Lymphoprep (StemCell Technologies, Vancouver, Canada) density gradient medium. PBMCs were washed twice in PBS, then stored cryopreserved in vapour phase liquid nitrogen for later use. PBMCs (1.1 × 10^6^) were plated into 96‐well plates for both assays as described previously.^68^, ^69^ For the activation‐induced marker (AIM) assay, PBMCs were stimulated in complete RPMI (cRPMI) medium [RPMI1640 medium (Invitrogen, Thermo Fisher Scientific)] supplemented with 2 mm L‐glutamine (Gibco), 1 mm MEM sodium pyruvate (Gibco), 100 μm MEM non‐essential amino acids (Gibco), 5 mm HEPES buffer solution (Gibco), 55 μm 2‐mercaptoethanol (Gibco), 100 U mL^−1^ penicillin (Gibco), 100 μg mL^−1^ streptomycin (Gibco) and 10% fetal bovine serum (Gibco) with SARS‐CoV‐2 Spike peptide pool [1 μg mL^−1^] [181 peptides, 0.06 mg mL^−1^ per peptide; BEI Resources, NIAID, NIH, SARS‐Related Coronavirus 2 Spike (S) Glycoprotein, NR‐5240] or dimethylsulfoxide [DMSO; negative control; 0.1%; Sigma‐Aldrich (Merck), Burlington, USA] and cultured at 37°C and 5% CO_2_ for 24 h. Cells were then washed and stained with CXCR5‐BV421 (562747; BD Biosciences), CD3‐BV510 (317332; BioLegend, San Diego, USA), CD8‐BV605 (564116; BD Biosciences), CD4‐BV650 (563875; BD Biosciences), CD25‐BV711 (563159; BD Biosciences), CXCR3‐BV786 (353738; BD Biosciences), CD137‐APC (309810; BioLegend), CD27‐AF700 (560611; BD Biosciences), CD14‐APC‐H7 (560180; BD Biosciences), CD19‐APC‐H7 (560252; BD Biosciences), Live/Dead NIR (L34976; Invitrogen), CD69‐PerCPCy5.5 (310925; BioLegend), CD134‐PE (340420; BD Biosciences), CD95‐PE‐CF594 (562395; BD Biosciences), CD45RA‐PeCy7 (337167; BD Biosciences) after which cells were fixed with 1% paraformaldehyde.

For the intracellular cytokine staining (ICS) assay, PBMCs were stimulated with 100 μg mL^−1^ SARS‐CoV‐2 Spike peptide pool (as detailed above) or DMSO (negative control; 0.1%; Sigma‐Aldrich) in combination with anti‐CD28/CD49d (1:100, 347690; BD Biosciences), 10 U mL^−1^ IL‐2 (11147528001, Roche) for 24 h at 37°C and 5% CO2, with brefeldin A (555029, BD Biosciences) added after 8 h. Cells were subsequently washed twice with MACS buffer (PBS, 0.5% BSA, 2 mM EDTA) and stained with surface antibodies CD3‐BV510, CD4‐BV650, CD8‐PerCPCy5.5 (565310; BD Biosciences) and Live/Dead NIR for 30 min on ice. Cells were washed twice and fixed using the BD Cytofix/cytoperm kit (554723; BD Biosciences) according to the manufacturer's instructions, followed by an intracellular staining with IFN‐γ‐v450 (560371; BD Biosciences) and TNF‐α‐AF700 (557996; BD Biosciences) for 30 min on ice. Cells were washed twice and resuspended in MACS buffer before acquisition on an LSR Fortessa II (BD Biosciences) followed by the analysis using the FlowJo software (v10).

### Statistical analysis

Statistical analysis was done using Stata version 18.0 (StataCorp LLC, College Station, USA) and R^70^ using R Studio (2022.07.1+54), and depicted graphically with R using R Studio (2022.07.1+54) and GraphPad Prism (version 9.1.0, GraphPad Software, Boston, USA). Participants were excluded from analysis if they had positive NCP serology at any time point.

For cytokine data, values below the lower limit of detection for each analyte were assigned a value of half the lowest detected value for that analyte and values above the upper limit of detection were excluded.^36^ For iSARS stimulation, the proportion of values above the upper limit of detection was IP‐10 0.1%, MCP‐1 6.9%. The remaining cytokines had all values below the upper limit of detection. For other stimuli, the proportion of samples within the limit of detection are presented in Supplementary table 2. Samples were excluded if blood was taken outside of the blood collection window (V0 = 1, V2 = 3). The effect of BCG vaccination on responses to iSARS stimulation was assessed using linear regression of log‐transformed cytokine data adjusted for: nil (iVero) stimulation cytokine response, sex, BCG vaccination prior to BRACE trial, BRACE trial stage (stage 1 or 2), days between randomisation and blood collection, age at first COVID‐19 vaccine dose (V1/V2) or V0 blood collection (V0 only), days between COVID‐19 vaccine dose and blood collection (V1/V2), days between first and second COVID‐19 doses (V2 only) batch of pre‐prepared stimulant and assay kit. Distribution of data for IL‐3 and IL‐7 remained non‐normal after log transformation; therefore, these iSARS responses to these cytokines were analysed by bootstrapped quantile regression of the raw cytokine data with adjustments as above for the linear regression.

Principal component analysis was performed with the prcomp function in R and was used to investigate variations in cytokine concentrations following iSARS stimulation at baseline (V0), post‐ChAdOx1‐S vaccination (V2) and post‐BNT162b2 vaccination (V2). Prior to PCA, cytokine concentrations were standardised using *z*‐score normalisation to ensure equal contribution to the analysis, irrespective of their original scales. The analysis evaluated the influence of participant characteristics as baseline (e.g. sex, obesity), technical variation and BCG vaccination status on the principal components. This influence was quantified using the coefficient of determination (*R* ^2^) values and associated *P*‐values, providing insights into the extent to which these factors explain the variance captured by each principal component. Additionally, the contribution (loading) of each cytokine to the principal components was calculated. Cytokines with the highest contributions to the principal components were highlighted, offering a focussed view on the cytokines most associated with the variance observed in response to iSARS stimulation under different vaccination conditions.

For multiplex serology data, differences before (V0) and after (V2) COVID‐19 vaccination were determined by Wilcoxon matched‐pairs signed rank test and differences between participants in the Control and BCG groups were determined by Wilcoxon rank‐sum test. For serology ELISA data, samples were excluded if blood was taken outside of the blood collection window (V0 = 6, V1 = 6, V2 = 9). Differences between participants in the Control and BCG groups were assessed using linear regression of log‐transformed antibody area under the curve (AUC) data adjusted for: sex, BCG vaccination prior to BRACE trial, BRACE trial stage (stage 1 or 2), days between randomisation and blood collection, age at first COVID‐19 vaccine dose (V2) or V0 blood collection (V0 only), days between COVID‐19 vaccine dose and blood collection (V2), days between first and second COVID‐19 doses (V2 only).

The gating strategy for the T‐cell activation assays, is presented in Supplementary figure 6. Differences before (V0) and after (V2) COVID‐19 vaccination and between DMSO and Spike stimulated T cells were determined by two‐way ANOVA with Tukey correction for multiple comparisons in GraphPad Prism. Differences between participants in the Control and BCG groups were assessed using bootstrapped quantile regression adjusted for: sex, BCG vaccination prior to BRACE trial, BRACE trial stage (stage 1 or 2), days between randomisation and blood collection, age at first COVID‐19 vaccine dose (V2) or V0 blood collection (V0 only), days between COVID‐19 vaccine dose and blood collection (V2), and days between first and second COVID‐19 doses (V2) in Stata.

## Author contributions

**Nicole L Messina:** Conceptualization; funding acquisition; methodology; formal analysis; investigation; data curation; project administration; supervision; visualization; writing – original draft. **Susie Germano:** Investigation; methodology; resources; writing – review and editing. **Amy W Chung:** Formal analysis; methodology; resources; writing – review and editing. **Carolien E van de Sandt:** Formal analysis; investigation; methodology; resources; writing – review and editing. **Natalie E Stevens:** Resources; writing – review and editing. **Lilith F Allen:** Investigation; writing – review and editing. **Rhian Bonnici:** Data curation; investigation; writing – review and editing. **Julio Croda:** Funding acquisition; writing – review and editing. **Claudio Counoupas:** Funding acquisition; writing – review and editing. **Branka Grubor‐Bauk:** Methodology; writing – review and editing. **Ebene R Haycroft:** Investigation; writing – review and editing. **Katherine Kedzierska:** Methodology; resources; writing – review and editing. **Ellie McDonald:** Data curation; investigation; writing – review and editing. **Rebecca McElroy:** Investigation; writing – review and editing. **Mihai G Netea:** Funding acquisition; writing – review and editing. **Boris Novakovic:** Formal analysis; funding acquisition; visualization; writing – review and editing. **Kirsten P Perrett**: Methodology; writing – review and editing. **Laure F Pittet:** Methodology; writing – review and editing. **Ruth A Purcell:** Investigation; writing – review and editing. **Kanta Subbarao:** Funding acquisition; methodology; resources; writing – review and editing. **James A Triccas:** Funding acquisition; writing – review and editing. **David J Lynn:** Funding acquisition; methodology; resources; writing – review and editing. **Nigel Curtis:** Conceptualization; funding acquisition; methodology; writing – review and editing.

## Conflict of interest

Outside the submitted work, JC has received grants or contracts from Valneva/Butantan, MSD, Sanofi Pasteur, CEPI/Sabin Institute, Takeda and NIH; payment or honoraria for presentations from Pfizer and is on the Brazil and/or Latin America Advisory Boards for Modern/Zodiac, Pfizer and Takeda. MGN is a scientific founder of Lemba, Biotrip and TTxD. All other authors declare no conflicts of interest.

## Ethics

The BRACE trial, which includes the BCOS sub‐study, was approved by the Royal Children's Hospital Human Research Ethics Committee (HREC) (No. 62586) with HREC and/or governance approval at all participating sites.

## Supporting information


**Supplementary** **figures** **1‐6**

**Supplementary** **tables** **1‐9**

## Data Availability

Deidentified analysis data and data dictionary are available to others upon request and on completion of a signed data access agreement. Requests can be made in writing to braceresearch@mcri.edu.au.
