# Supplementary material for: Effect of Bacille Calmette–Guérin vaccination on immune responses to SARS‐CoV‐2 and COVID‐19 vaccination
Source: Clin Transl Immunology. 2025 Jan 25;14(1):e70023. doi: 10.1002/cti2.70023 (PMC11761716; doi:10.1002/cti2.70023)
Supplement: Supplementary file 1 — Supplementary figures 1‐6 [file CTI2-14-e70023-s001.pdf]

Supplementary figure 1

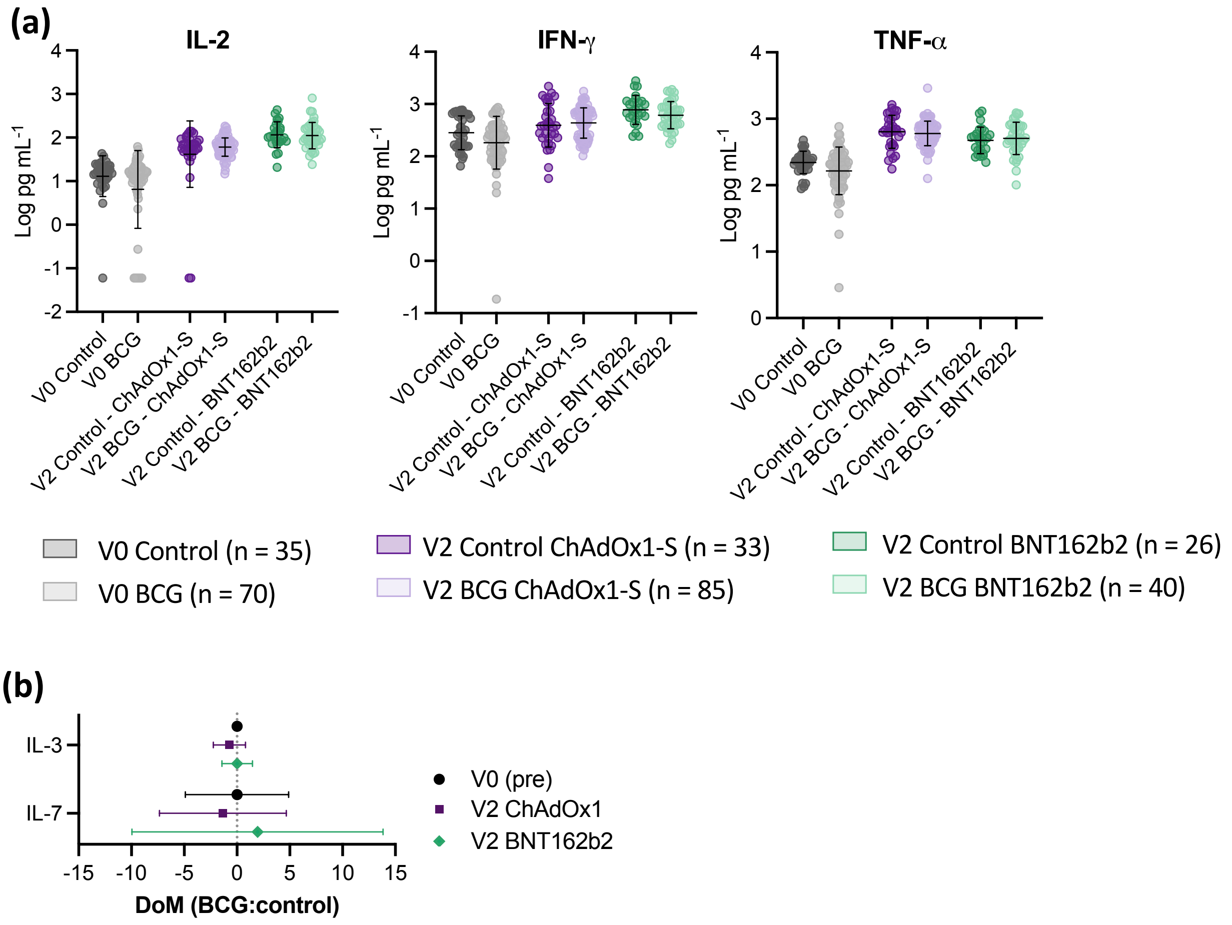

Supplementary figure 1

Whole blood cytokine responses to γ-irradiated SARS-CoV-2 Blood samples were taken before (V0) and 28 days after the second vaccination dose (V2) of COVID-19 vaccinations. **(a)** Scatter dotplots depicting log-transformed cytokine responses. Line represents the mean and errors bars are standard deviation. **(b)** Forest plots depicting adjusted difference of medians (DoM) and 95% confidence intervals for the effect of BCG vaccination determined by multivariable quantile regression. DOM > 0 indicates responses that were higher for BCG-vaccinated compared to Control participants.

Supplementary figure 2

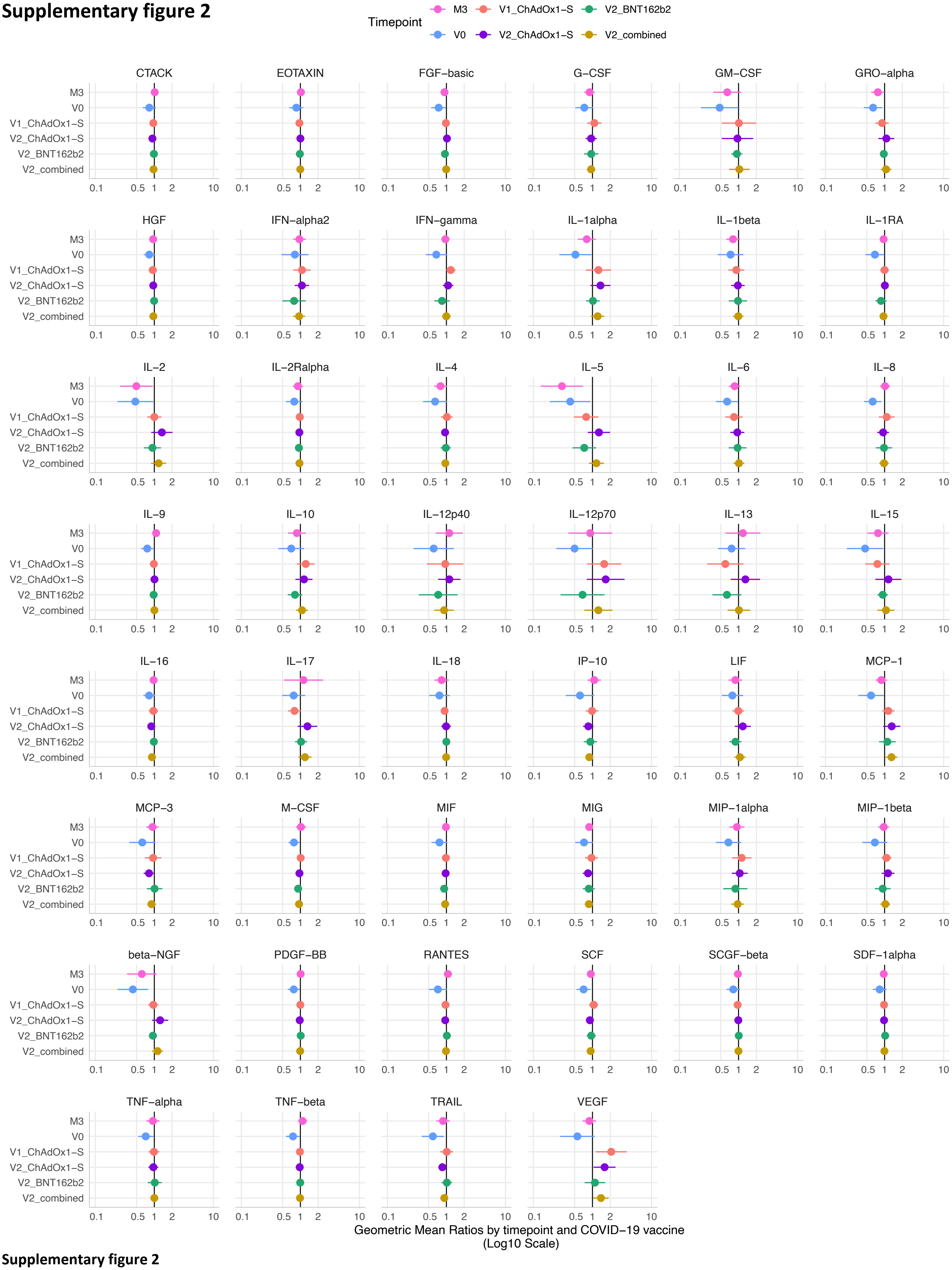

Supplementary figure 2

Whole blood cytokine responses to γ-irradiated SARS-CoV-2 in blood samples taken 3-months post randomisation (M3, Control = 92, BCG = 130), before COVID-19 vaccinations (V0, Control = 35, BCG = 70), 28 days after the first ChAdoX1-S dose (V1_ChAdoX1-S, Control = 29, BCG = 61), and 28 days after the second dose of ChAdoX1-S (V2_ChAdoX1-S, Control = 33, BCG = 85), BNT162b2 (V2_BNT162b2, Control = 26, BCG = 40) or either COVID-19 vaccine (V2_combined). Forest plots depict the adjusted geometric mean ratios (GMR) and 95% confidence intervals for the effect of BCG vaccination determined by multivariable linear regression. GMR > 1.0 indicates responses that were higher for the BCG compared to Control Group. *P < 0.05.

Supplementary figure 3

(a)

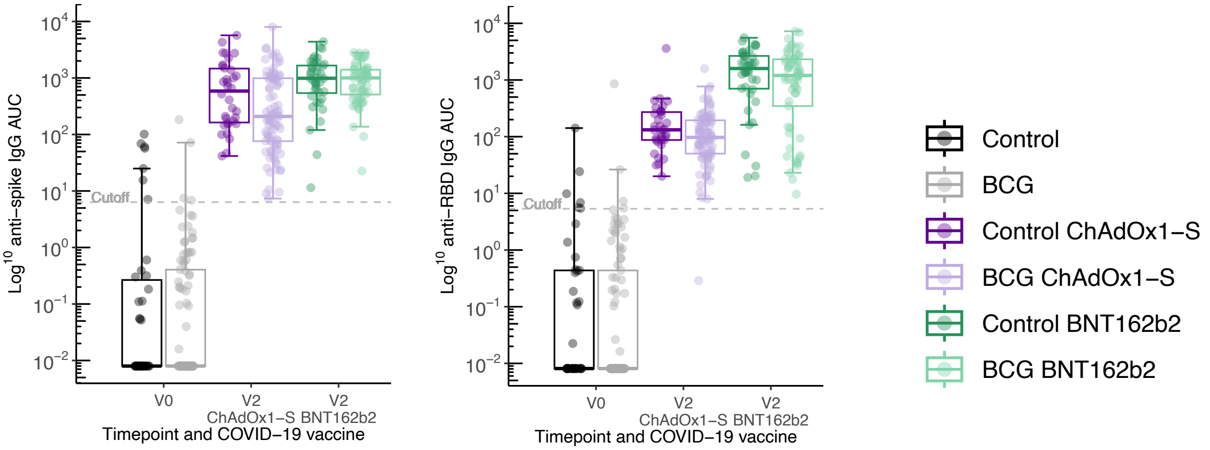

(b)

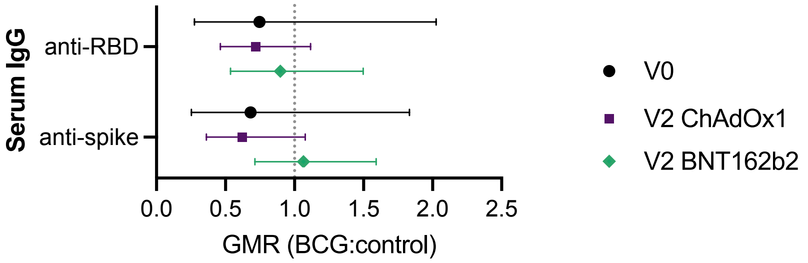

Supplementary figure 3

(a) Boxplot and scatter dotplot presenting anti-spike and anti-RBD IgG AUC before (V0, n = 133) and 28 days after the second dose of COVID-19 vaccination (V2; ChAdOx1-S n = 124; BNT162b2, n = 101) in BCG vaccinated and Control participants. **(b)** Forest plots depicting adjusted geometric mean ratios (GMR) and 95% confidence intervals for the effect of BCG vaccination on anti-spike and anti-RBD IgG AUC at V0 and V2 determined by multivariable linear regression. GMR > 1.0 indicates responses that were higher for BCG-vaccinated compared to Control participants. Data were log-transformed prior to analysis. *P*-value < 0.05 depicted by *.

Supplementary figure 4

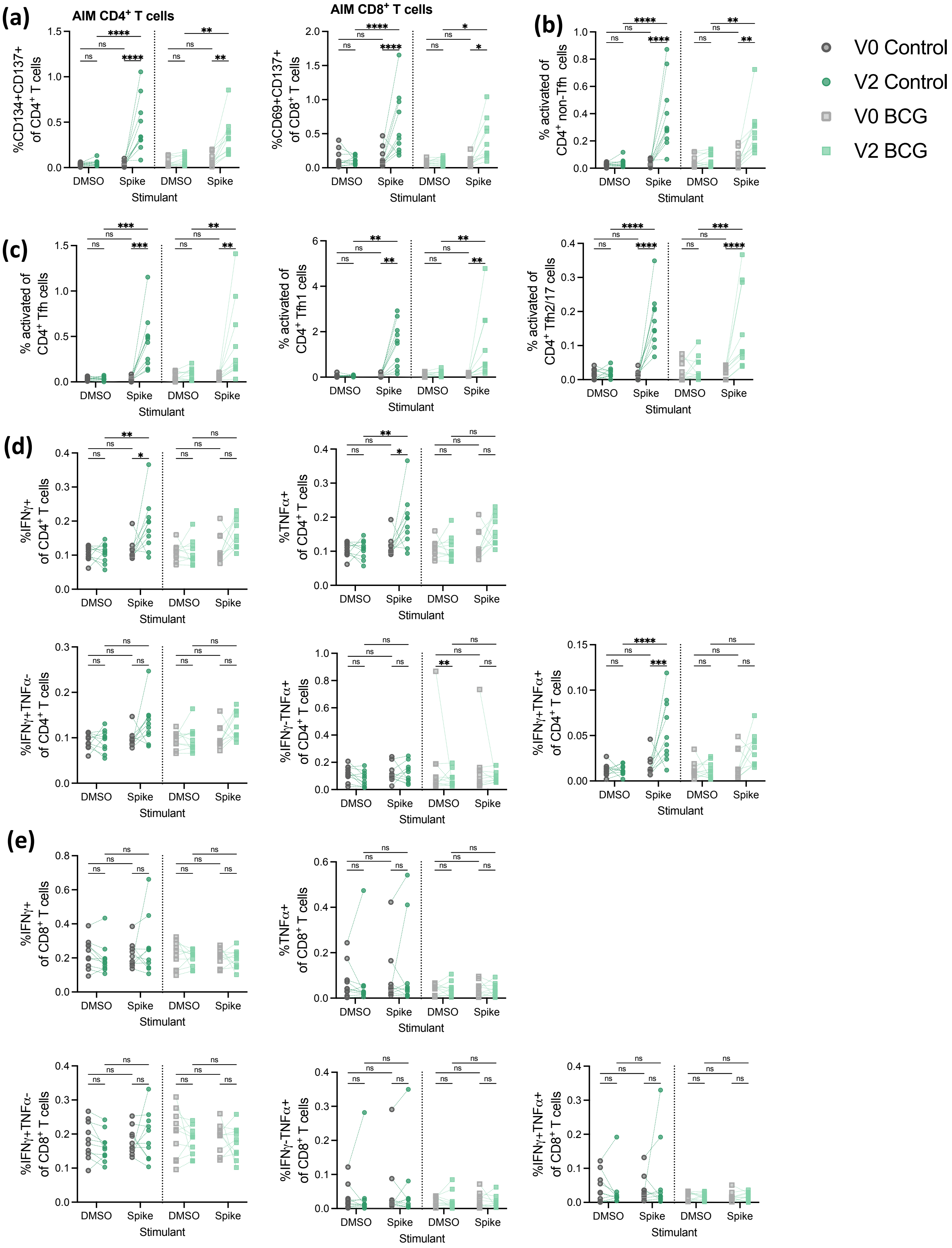

Supplementary figure 4

Before-and-after dotplots depicting changes in (**a-b, d**) CD4$^+$ T cell, (**c**) T follicular helper (Tfh) cell and (**a, e**) CD8$^+$ T cell (**a-c**) activation and (**d-e**) cytokine production in DMSO and Spike stimulated PBMCs before (V0) and 28 days after the second dose of BNT162b2 (V2) in BCG-vaccinated (n = 10) and Control (n = 10) participants. Difference before (V0) and after (V2) COVID-19 vaccination were determined by Wilcoxon matched-pairs signed rank test *$P < 0.05$, **$P < 0.01$, ***$P < 0.001$, ****$P < 0.0001$, ns $P \geq 0.05$.

Supplementary figure 5

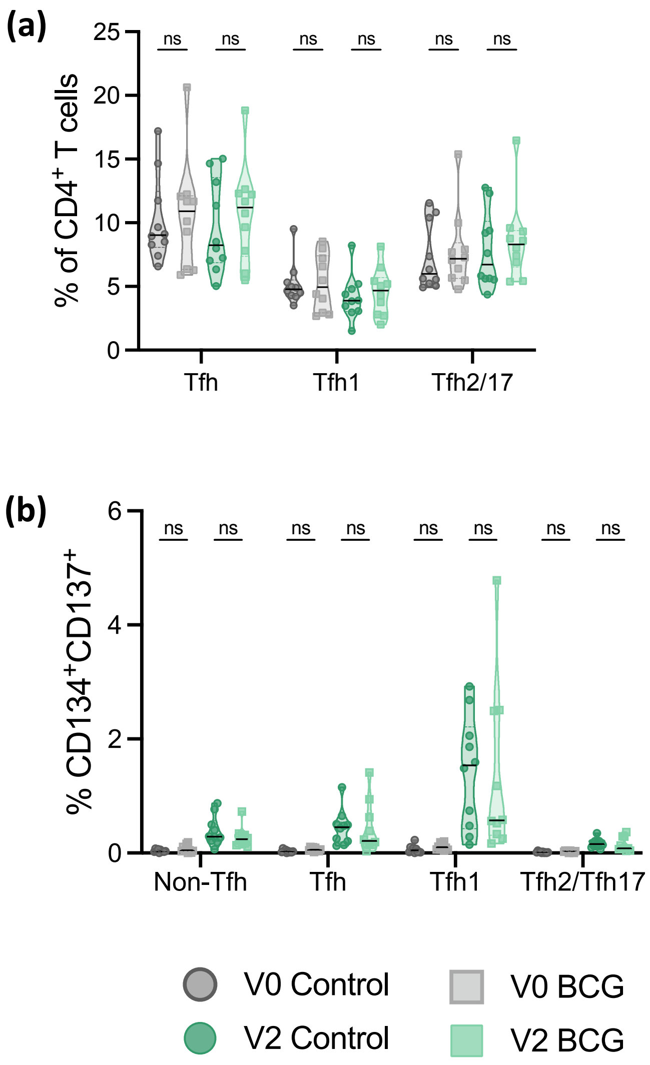

Supplementary figure 5

Violin graph with scatter dot-plot presenting **(a)** the percentage T follicular helper (Tfh) cells among CD4⁺ T cells and **(b)** the percentage of activated (CD134⁺CD137⁺) cells within CD4⁺ T cell subsets. Differences between BCG-vaccinated (■ n = 10) and Control (● n = 10) participants were determined by bootstrapped quantile regression. Abbreviations: BCG, bacille Calmette Guérin; ns, non-significant (*P* ≥0.05); Tfh, T follicular helper; V0, before COVID-19 vaccination; V2, 28 days after COVID-19 vaccination

Supplementary figure 6

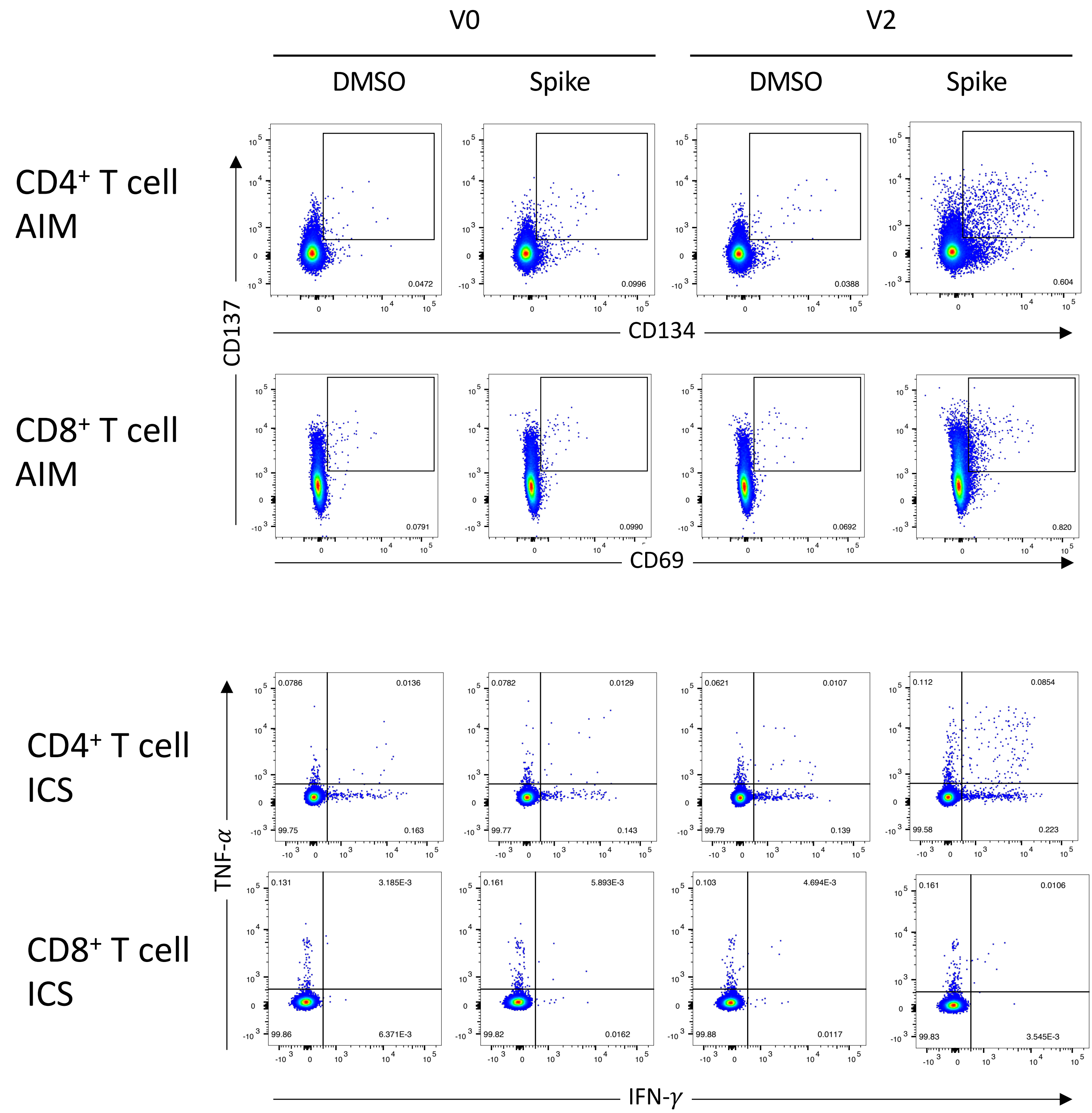

Supplementary figure 6
Activation induced marker (AIM) and intracellular cytokine staining (ICS) gating strategy.
